# Evolutionary conservation of the fidelity of transcription

**DOI:** 10.1038/s41467-023-36525-w

**Published:** 2023-03-20

**Authors:** Claire Chung, Bert M. Verheijen, Zoe Navapanich, Eric G. McGann, Sarah Shemtov, Guan-Ju Lai, Payal Arora, Atif Towheed, Suraiya Haroon, Agnes Holczbauer, Sharon Chang, Zarko Manojlovic, Stephen Simpson, Kelley W. Thomas, Craig Kaplan, Peter van Hasselt, Marc Timmers, Dorothy Erie, Lin Chen, Jean-Franćois Gout, Marc Vermulst

**Affiliations:** 1 School of Gerontology, University of Southern California, Los Angeles, CA USA; 2 Department of Biological Sciences, University of Pittsburgh, Pittsburgh, PA USA; 3 Children’s hospital of Philadelphia, Center for Mitochondrial and Epigenomic Medicine, Philadelphia, PA USA; 4 Keck School of Medicine, University of Southern California, Los Angeles, CA USA; 5 College of Life Sciences and Agriculture, University of New Hampshire, Durham, NH USA; 6 Department of Metabolic Disease, University of Utrecht, Utrecht, the Netherlands; 7 Department of Urology, Medical Center - University of Freiburg, Freiburg, Germany; 8 German Cancer Consortium (DKTK) Partner Site Freiburg, German Cancer Research Center (DKFZ), Heidelberg, Germany; 9 Department of Chemistry, University of North Carolina, Chapel Hill, NC USA; 10 Department of Molecular and Cellular Biology, University of Southern California, Los Angeles, CA USA; 11 Department of Biological Sciences, Mississippi State University, Mississippi State, MS USA

**Keywords:** Gene expression, Mechanisms of disease, Evolutionary genetics, Molecular evolution

## Abstract

Accurate transcription is required for the faithful expression of genetic information. However, relatively little is known about the molecular mechanisms that control the fidelity of transcription, or the conservation of these mechanisms across the tree of life. To address these issues, we measured the error rate of transcription in five organisms of increasing complexity and found that the error rate of RNA polymerase II ranges from 2.9 × 10^−6^ ± 1.9 × 10^−7^/bp in yeast to 4.0 × 10^−6^ ± 5.2 × 10^−7^/bp in worms, 5.69 × 10^−6^ ± 8.2 × 10^−7^/bp in flies, 4.9 × 10^−6^ ± 3.6 × 10^−7^/bp in mouse cells and 4.7 × 10^−6^ ± 9.9 × 10^−8^/bp in human cells. These error rates were modified by various factors including aging, mutagen treatment and gene modifications. For example, the deletion or modification of several related genes increased the error rate substantially in both yeast and human cells. This research highlights the evolutionary conservation of factors that control the fidelity of transcription. Additionally, these experiments provide a reasonable estimate of the error rate of transcription in human cells and identify disease alleles in a subunit of RNA polymerase II that display error-prone transcription. Finally, we provide evidence suggesting that the error rate and spectrum of transcription co-evolved with our genetic code.

## Abstract (web summary)

The molecular mechanisms that ensure faithful transcription of genetic information are still unclear. Chung et al. identify various genes, alleles and processes that affect the fidelity of transcription multiple organisms, suggesting evolutionary conservation of fidelity factors, and compare the error rate of transcription among these species.

## Introduction

The genome provides a precise, biological blueprint of life. To preserve this blueprint for future generations, organisms replicate their genome with remarkable precision^1^. This precision relies on multiple safety mechanisms that are built into the DNA replication machinery itself^1,2^, as well as hundreds of proteins that improve the fidelity of DNA replication through a wide variety of other mechanisms. For example, DNA repair proteins^3^, epigenetic regulators^4^, translesion polymerases^5^, topoisomerases^6^, the nuclear envelope^7^ and numerous proteins that control the order with which origins of DNA replication fire^8^ all contribute to the fidelity of DNA replication through their own, unique mechanisms. In human cells, these mechanisms ensure that as few as ∼1.5 mutations arise per cell division^9^, or 60 mutations per generation^10^. In addition, it is important that the genome is accurately expressed. But compared to DNA replication, relatively little is known about the mechanisms that ensure the fidelity of gene expression. Our most detailed knowledge of these mechanisms comes from structural and biochemical studies of RNAPII^11–20^. These studies show that like DNA polymerases, RNA polymerases contain an intrinsic set of safety mechanisms that allow RNAPII to select the correct nucleotides, prevent mismatches from being extended, and mismatched nucleotides to be excised after misincorporation^15^. Because these parameters differ from mismatch to mismatch, the error rate of each misincorporation event is a unique composite of these three variables^12^.

In addition, these safety mechanisms provide a platform for proteins associated with the RNAPII holoenzyme to promote accurate transcription. For example, it was previously shown in yeast that the TFIIS protein improves the fidelity of transcription by promoting the ability of RNAPII to excise misincorporated nucleotides^21–24^. Reduced excision of mismatched nucleotides is also a feature of cells that lack Rpb9^25^, a small subunit of the RNAPII holoenzyme that plays a key role in the fidelity of transcription in yeast. In addition, Rpb9 promotes accurate transcription by improving the selection of complementary nucleotides^26,27^ and limiting the extension of mismatched bases^25^. One mechanism by which Rpb9 is thought to accomplish these tasks, is through its interactions with the trigger loop, a highly dynamic structure of RNAPII that opens and closes during each incorporation cycle^26^. Several mutations in this structure (including the yeast Rpb1^E1103G^ mutation) are known to result in error-prone transcription by allowing mismatched bases to remain in the active site longer than normal, which facilitates misincorporation^16^.

These safety mechanisms can also be affected by environmental factors. For example, some environmental mutagens change the chemistry of DNA in such a way that its base-pairing properties are fundamentally altered^28–32^. Because RNAPII depends on these properties to select the correct nucleotides and make decisions about the extension and excision of misincorporated bases^33^, DNA damage allows mismatched bases to circumvent the inherent safety mechanism of RNAPII^34,35^.

Here, we build on these findings to fill three major gaps in our knowledge. First, most studies on the fidelity of transcription focus on the fidelity of RNAPII. As a result, relatively little is known about the fidelity of RNAPI, RNAPIII or the mitochondrial RNA polymerase. Because these enzymes are essential components of the central dogma of life as well, it will be important to understand how these enzymes maintain accurate transcription. Second, most fidelity experiments (although not all^21,28,30,31,36,37^) have been performed in vitro, with various combinations of isolated proteins and synthetic templates, often with the help of crystal structures or cryoEM. It is often difficult to translate these findings to living organisms, where countless proteins interact with the transcription machinery, and a complex chromatin landscape needs to be navigated in a highly dynamic environment that could modulate the accuracy of RNA synthesis in numerous ways. A third issue is that most experiments, including our own, have been performed in micro-organisms, or proteins that were derived from micro-organisms. Due to technical limitations, it has been difficult to perform similar experiments in multicellular organisms, so that it is frequently unknown if the fidelity factors and alleles discovered in bacteria or yeast cells are functionally conserved in higher organisms as well.

To address these issues, and combine the knowledge gained from structural studies with measurable phenotypes, we used a massively parallel sequencing assay to monitor the fidelity of transcription in 5 organisms of increasing cell number, cell type and body size. These organisms are the budding yeast *Saccharomyces cerevisiae*, the nematode *Caenorhabditis* *elegans*, the fruitfly *Drosophila* *melanogaster* and primary cells derived from mice and humans. Our sequencing technology surveys the entire transcriptome for transcription errors, so that the fidelity of every major RNA polymerase could be determined. We then deleted, mutated or knocked down multiple subunits of these polymerases in yeast to identify proteins and alleles that play a role in the fidelity of transcription and examined how well the strongest fidelity factors are conserved between species. These findings ultimately led us to investigate 2 cohorts of human patients that carry mutations in subunits of RNAPII, which allowed us to identify patient-specific alleles that result in error-prone RNA polymerases. In addition, we used this information to determine the fidelity of transcription in human cells.

## Results

### Multiple proteins are required to maintain the fidelity of transcription in yeast

**Fig. 1:**
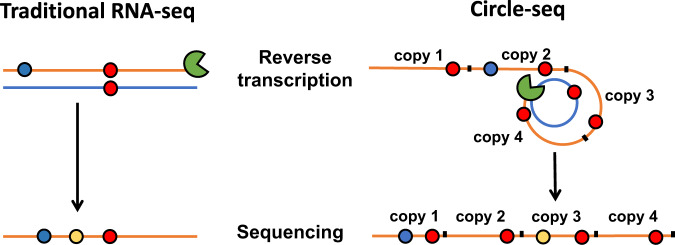
Core concept of the circle-sequencing assay. Traditional sequencing approaches can identify transcription errors (red circle) present in RNA molecules (blue lines). However, during reverse transcription of RNA templates, additional errors (blue circles) are introduced into the cDNA (yellow lines) by reverse transcriptases that are indistinguishable from true transcription errors. Additional artifacts (yellow circles) that resemble transcription errors are also introduced during sequencing itself. To prevent these artifacts from confounding our measurements, RNA is circularized prior to reverse transcription. These circularized molecules are then reverse transcribed in a rolling circle fashion to generate linear cDNA molecules constructed of tandem repeats of the original RNA fragment. If a transcription error was present in the original template, this error will be present in all copies of this template, while artifacts will occur only once.

**Fig. 2:**
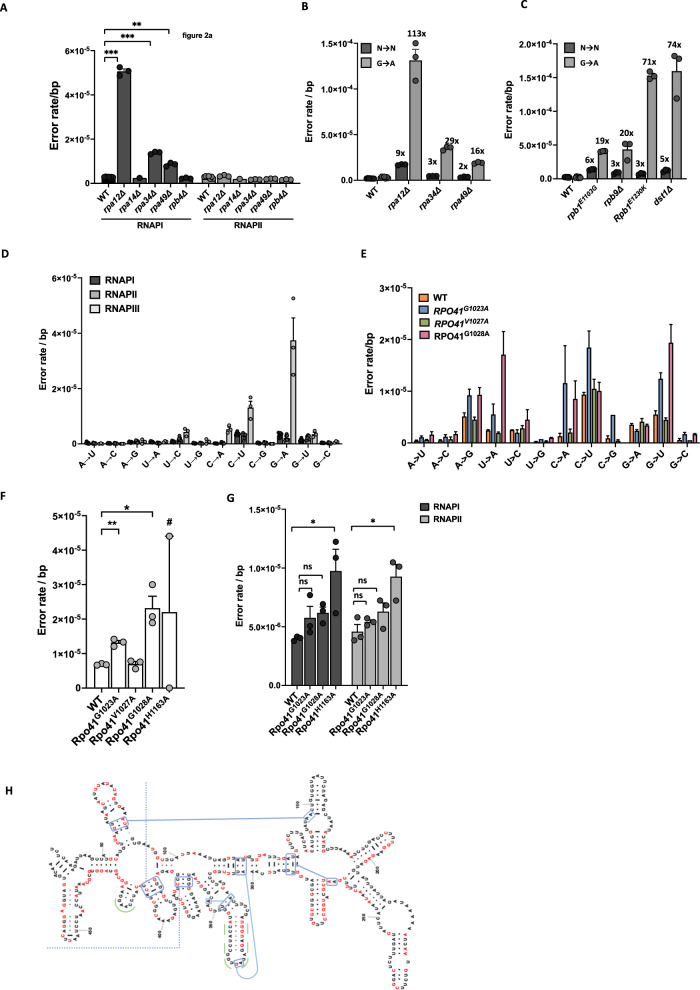
A survey of proteins and alleles that control the fidelity of transcription in yeast. **A** Rpa12 (*n* = 3, *P* = 0.0005), Rpa34 (*n* = 3, *P* = 0.0005) and Rpa49 (*n* = 3, P = 0.0061) are required for high-fidelity transcription by RNAPI (WT *n* = 7, Rpa14 *n* = 1)**. B**, **C** All the error-prone alleles identified so far in RNAPI and II specifically increase G→A errors (*n* identical to **A**). **D** RNAPIII displays a higher error rate than RNAPI and II primarily due to an increased G→A error rate (*n* = 7). **E** The error spectrum of WT and Rpo41 mutants (*n* = 3). **F** Two alleles, *Rpo41*^*G1023A*^ (*P* = 0.0092) and *Rpo41*^*G1028A*^ (*P* = 0.01) display increased error rates (*n* = 3). A third allele, *Rpo41*^*H1163A*^ resulted in too few reads from the mitochondrial genome to draw firm conclusions (#). **G** Cells that carry the *Rpo41*^*H1163A*^ allele display an increased error rate in nuclear RNA (*n* = 3, *P* = 0.0344 for RNAPI and *P* = 0.255). **H** Transcription errors detected in18S rRNA. Red bases indicate bases in which errors were detected. Green and blue lines indicate that these bases form secondary structures or make connections with other rRNA subunits. The errors we detected affect every aspect of RNA structure and function. In all cases, *n* is defined as biologically independent replicates. All experiments were analyzed by unpaired, two-tailed Welch’s *t*-tests using Prism software. *P < 0.05, **P < 0.01, ***P < 0.001. Error bars depict standard error from the mean. Source data are provided as a Source Data file.

Using genetically engineered yeast strains^21,36,37^ and massively parallel sequencing technology^34,35^, we and others previously demonstrated that the non-essential subunits Rpb9 and TFIIS enhance the fidelity of transcription of RNAPII in living cells^16,21,26,34–39^. In addition, we showed that the functional analog of these proteins, Rpa12, controls the in vivo fidelity of RNAPI in yeast^35^. To further unravel the molecular architecture that controls the fidelity of transcription, we investigated whether the remaining non-essential subunits of RNAPI and II also play a role in the fidelity of transcription. First, we measured the error rate of yeast cells that carry a deletion of Rpa14, Rpa34 or Rpa49, the three remaining non-essential subunits of RNAPI. To do so, we used an optimized version of the circle-sequencing assay^35,40^, a consensus sequencing approach that was initially designed to measure the mutation rate of RNA viruses^41,42^ (Fig. 1). These experiments revealed that loss of Rpa34 and Rpa49 increased the base substitution rate of RNAPI ∼4-fold (1.4 × 10^−5^ ± 6.3 × 10^−7^/bp for *Rpa34Δ* cells and 8.6 × 10^−6^ ± 7.8 × 10^−7^/bp for *Rpa49Δ* cells, Fig. 2A), while the rate of insertions and deletions increased 2-fold (Fig. S1a, b), indicating that both Rpa34 and Rpa49 are important for the fidelity of RNAPI in living yeast. Full tables containing the number of errors detected and bases sequenced for key datapoints are listed in supplementary table 1. Interestingly, recent in vitro experiments directly support this conclusion^43^. We further note that no increase was detected in the error rate of RNAPII, consistent with the idea that Rpa34 and Rpa49 are only part of the RNAPI holoenzyme. In addition, these observations serve as an internal control to demonstrate that the reduced fidelity of RNAPI was not the result of unrelated, cellular conditions that affected transcriptional fidelity. In contrast, loss of Rpa14 had no effect on the error rate of transcription, indicating that it does not play a role in transcriptional fidelity. When we examined the increased error rates of *Rpa34Δ* and *Rpa49Δ* cells further, we found that they were primarily fueled by an increase in G→A errors (Fig. 2B). Although other errors increased in abundance as well (full error spectra are depicted in Figs. S2 and S3), a disproportionate increase in G→A errors characterized every other mutant yeast strain known to display error-prone transcription, including *Rpa12Δ*, *Rpb9Δ, Dst1Δ* (*DST1* encodes TFIIS), and *Rpb1*^*E1103G*^ cells^35^ (RNAPII, Fig. 2C). This comparison suggests that G→A errors pose a substantial threat to the fidelity of transcription and that multiple proteins evolved to prevent them. Interestingly, loss of the fidelity factor GreA increases the rate of G→A errors in bacteria as well, further underlining the universality of this observation^44^.

Next, we examined the error rate of yeast cells that lack Rpb4, the only non-essential subunit of RNAPII in addition to Rpb9 and TFIIS, and a functional homolog of Rpa14. Like Rpa14, loss of Rpb4 had no effect on the error rate of RNAPI or II (Fig. 2A), suggesting that it does not play a role in transcriptional fidelity. In addition to non-essential subunits, essential components of RNAPII could contribute to the fidelity of transcription as well. To examine this possibility, we analyzed yeast strains from the DamP collection^45^, which carry insertions in the 5′UTR of essential genes that reduce their expression (Table 1). From this collection, we analyzed strains with reduced expression of *Rpb3*, *Rpb5, Rpb7*, and *Rpb8*, all of which encode core components of the RNAPII holoenzyme. In addition, we tested whether reduced expression of *Tfg1* or *FcpI* affects the fidelity of RNAPII. Tfg1 and FcpI are part of the TFIIF elongation complex and play a similar role in RNAPII as Rpa34 and Rpa49 in RNAPI, which suggests that they could affect transcriptional fidelity as well. However, all these strains displayed similar error rates compared to WT cells (Fig. S4). Although this result suggests that these proteins do not contribute to fidelity, it should be noted that the degree to which some genes were knocked down was insufficient to draw firm conclusions (Table 1). Another caveat of this experiment is that RNAPII holo-enzymes assembled without these proteins may not produce enough transcripts to affect the overall error rate of transcription. Accordingly, we propose that experiments with targeted point mutations that leave the initiation, processivity and elongation rate of RNAPII intact may be needed to resolve these issues. Similar experiments could also address whether auxiliary components of the transcription machinery, including those that are only transiently associated with this process, such as transcription coupled DNA repair proteins, alter the fidelity of transcription. Depending on the safety net these proteins affect, these mutations could alter the fidelity of transcription in various ways. Finally, these experiments could reveal whether residues that maintain the fidelity of transcription of RNAPII serve a similar function in related polymerases. For example, the *rpb1*^*E1103G*^ mutation causes error-prone transcription by RNAPII in yeast. It would be interesting to test whether the analogous mutation in RNAPI (Rpa190^E1124G^)^46^ plays a similar role in transcriptional fidelity.

**Table 1:** Yeast proteins and alleles monitored for their contribution to transcriptional fidelity

Protein/allele	RNA polymerase	Knock out/in/down	Error prone	Primary error
Rpa12	RNAP I	KO	Yes	G → A
Rpa14	RNAP I	KO	No	C → U
Rpa34	RNAP I	KO	Yes	G → A
Rpa49	RNAP I	KO	Yes	G → A
Rpb1^E1103G^	RNAP II	KI	Yes	G → A
Rpb1^E1230K^	RNAP II	KI	Yes	G → A
Rpb3	RNAP II	5.6-fold KD	No	C → U
Rpb5	RNAP II	1.1-fold KD	No	C → U
Rpb7	RNAP II	3.0-fold KD	No	C → U
Rpb8	RNAP II	2.2-fold KD	No	C → U
Rpb9	RNAP II	KO	Yes	G → A
TFIIS	RNAP II	KO	Yes	G → A
Tfg1	RNAPII	2.4-fold KD	No	C → U
Fcp1	RNAPII	1.2-fold KD	No	C → U
Rpc11	RNAP III	1.1-fold KD/het. KO	No	G → A
Rpo41^G1023A^	mtRNAP	KI	No	C → U
Rpo41^V1027A^	mtRNAP	KI	No	C → U
Rpo41^G1028A^	mtRNAP	KI	Yes	G → U
Rpo41^H1162A^	mtRNAP	KI	Yes	G → A/G → U

If a knock down was attempted of a protein with a DamP strain, the fold knockdown compared to WT cells is depicted in column 3. KD indicates that the protein of interest was knocked down, KI indicates that a mutant allele was knocked in to replace the endogenous gene, and KO indicates that a gene was knocked out by deletion of the entire open reading frame. Het KO indicates heterozygous knockout.

Since Rpa12, Rpb9, and TFIIS are the strongest modulators of transcriptional fidelity identified thus far, we investigated whether the functional analog of these proteins in RNAPIII (Rpc11) controls the in vivo fidelity of RNAPIII in yeast. Previous in vitro studies have shown that Rpc11 contains a strong, intrinsic cleavage activity that could improve the fidelity of RNAPIII as much as 1000-fold^18^. Unlike Rpa12, Rpb9 and TFIIS though, Rpc11 is essential for the viability of yeast cells, preventing examination of homozygous *Rpc11Δ* strains. Neither a DamP strain or a heterozygous knockout of Rpc11 displayed increased error rates though (Fig. S5), suggesting that specific mutations in Rpc11 that knock out its proofreading ability (including the Rpc11^E92H^ mutation^47^) may be needed to assess the role of Rpc11 on the fidelity of transcription in living cells. Our experiments did demonstrate though, that RNAPIII commits ∼5 times more mistakes than RNAPI and II (Fig. 2D), primarily due to an elevated G→A error rate. This phenotype strongly resembles the fidelity of RNAPI and II without its fidelity factors, suggesting that the cleavage activity of Rpc11, despite its potency, is still insufficient to fully suppress transcriptional mutagenesis in living cells.

Finally, we examined the fidelity of the mitochondrial RNA polymerase (Rpo41), a single subunit enzyme that resembles the T7 phage RNA polymerase. Four amino acids were previously described that control the fidelity of the phage polymerase^48^, suggesting that these residues could modulate the error rate of Rpo41as well. Therefore, we genetically engineered 4 analogous mutations into *Rpo41* by gene substitution, creating the *rpo41*^*G1023A*^, *rpo41*^*V1027A*^, *rpo41*^*G1028A*^ and *rpo41*^*H1163A*^ alleles, Table 1, Fig. S6a) and measured the error rates of these cell lines. Interestingly, we found that cells that carry the *rpo41*^*G1023A*^ and *rpo41*^*G1028A*^ mutations indeed displayed an elevated error rate in mitochondrial RNA (1.31 × 10^−5^ ± 1.05 × 10^−6^/bp for *rpo41*^*G1023A*^ cells and 2.32 × 10^−5^ ± 5.94 × 10^−6^/bp for *rpo41*^*G1023A*^ cells, Fig. 2E, F), demonstrating that the fidelity function of these amino acids is functionally conserved. In contrast to the error-prone RNAPI and II lines though, these error-prone lines did not display an elevated G→A error rate, consistent with the distinct evolutionary origins of Rpo41 compared to RNAPI and II. In contrast, the *rpo41*^*V1027A*^ allele did not affect the fidelity of mtRNA, indicating that not all T7 phage allelic affects are conserved in Rpo41. And finally, we were unable to accurately measure the fidelity of the *rpo41*^*H1163A*^ allele. Cells that carried this allele had lost the vast majority of their mtDNA and mtRNA (Fig. S6b), preventing confident measurements of RNA integrity. Because the mitochondrial RNA polymerase is required to generate RNA primers for mtDNA replication^49,50^, one explanation for this observation is that the *rpo41*^*H1163A*^ allele inhibits either the initiation, elongation, or processivity of Rpo41, thereby preventing the primers required for mtDNA replication to be generated efficiently.

We were surprised to find though, that *rpo41*^*H1163A*^ cells do display an increased error rate in nuclear RNA (Fig. 2g). Thus, there seems to be an unexpected relationship between mitochondrial function and the fidelity of transcription in the nucleus. Because oxidative damage is a powerful source of transcription errors^28–30^, it is possible that the *rpo41*^*H1163A*^ allele elevates the error rate of transcription in the nucleus by inducing reactive oxygen species. Alternatively loss of mtDNA could affect nuclear transcriptional fidelity by altering the production of mitochondrial iron-sulfur clusters, which is required for efficient DNA repair^51^ and potentially transcription^52^. Regardless, these experiments identify multiple alleles and proteins that play a role in transcriptional fidelity and in doing so, mutant strains that can now be used to understand the impact of transcription errors on cellular health, including errors that occur in mitochondrial RNA and rRNA. For example, our experiments show that transcription errors can affect every aspect of rRNA structure and function (Fig. 2H). How these errors affect cellular function can now be determined with the help of these mutant strains.

### Evolutionary conservation of fidelity genes in higher organisms

**Fig. 3:**
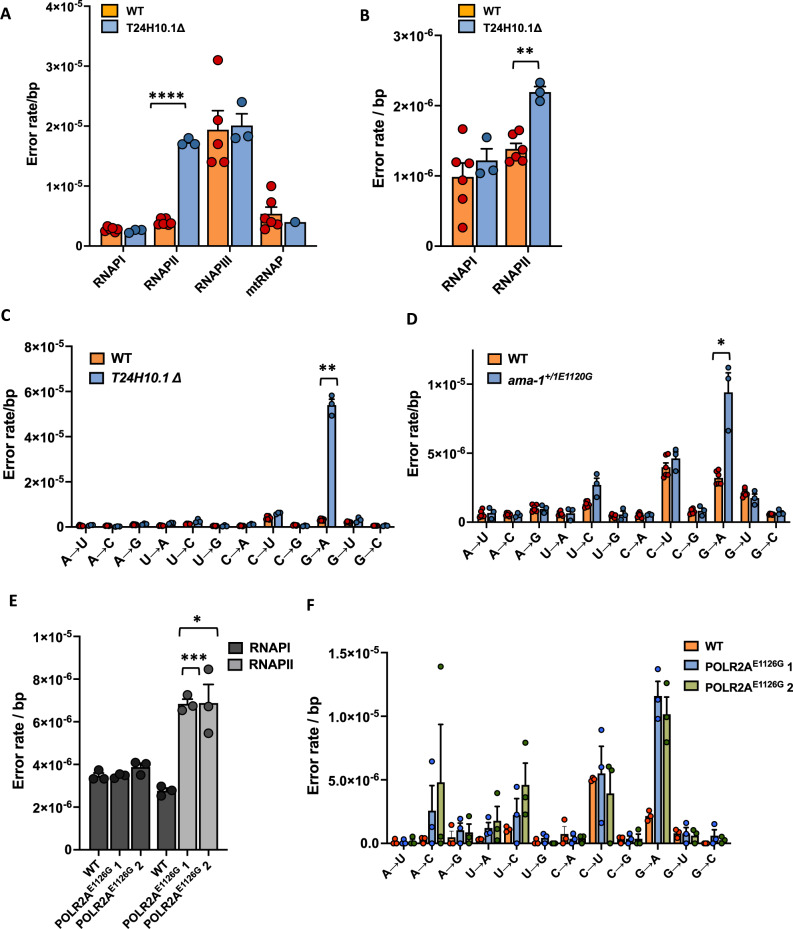
The error rate of transcription in genetically engineered worms and human cells. **A** Worms that carry a partial deletion in the T24H10.1 gene (*n* = 3, *P* < 0.0001) display an increased error rate in RNAPII but not RNAPI, III or the mitochondrial RNA polymerase compared to WT worms (*n* = 6). **B** RNAPII in T24H10.1Δ worms (n = 3) display an increased insertion rate compared to WT worms (*n* = 6, *P* = 0.0004). **C** The increased error rate of T24H10.1Δ worms (*n* = 3) is primarily fueled by G→A errors (*P* = 0.0025). **D** Worms that carry a heterozygous mutation in the *ama-1* gene (*ama-1*^*+/E1120G*^, *n* = 3) display an increased G→A error rate (*P* = 0.0458). **E** RNAPII in human cells that carry a homozygous mutation in the POLR2A gene (*POLR2A*^*E1126G*^, *n* = 3) displays an increased error rate compared to WT cells (*n* = 3), while RNAPI does not (*P* = 0.0003 for clone 1 and *P* = 0.0392 for clone 2). **F** The increased error rate of POLR2A^E1126G^ cells is primarily fueled by an increased G→A error rate. In all cases, *n* is defined as biologically independent replicates. All experiments were analyzed by unpaired, 2-tailed Welch’s t-tests using Prism software. *P < 0.05, **P < 0.01, ***P < 0.001. Error bars depict standard error from the mean. Source data are provided as a Source Data file.

Next, we wondered whether the fidelity factors identified in yeast play a similar role in multi-cellular organisms. To test this hypothesis, we selected the strongest fidelity factor in yeast (TFIIS) as our primary target and asked if TFIIS has a functional homolog in the nematode *C. elegans*. By searching for a similar protein sequence in the worm proteome with BLASTP we identified T24H10.1 as its closest relative (*E*-value 4 × 10^−27^), a protein that has with 27.5% identity and 54% strong similarity as determined by the Clustal Omega Alignment tool (Fig. S7). The only other close relative was a region of rpc-11, a subunit of RNAPIII in *C. elegans* that encodes an embedded TFIIS-like domain (*E*-value 2 × 10^−6^). We then examined a strain that contains a deletion in T24H10.1 and found that these worms display a 5-fold increase in base substitutions (1.72 × 10^−5^ ± 5.87 × 10^−7^/bp, Fig. 3A) and a 1.5-fold increase in insertions compared to control worms (Fig. 3B). Further analysis showed that the elevated error rate of *T24H10.1Δ* worms is fueled by G→A errors and that these errors are only increased in RNAs generated by RNAPII (Fig. 3A), suggesting that T24H10.1 is a fidelity factor for RNAPII (Fig. 3C). Thus, T24H10.1Δ worms display a similar fidelity defect compared to TFIIS null cells, indicating that T24H10.1 is indeed a functional homolog of TFIIS in *C. elegans*. This strain provides a unique opportunity to understand the impact of transcription errors on organismal health.

Next, we wondered whether the fidelity effect of specific amino acids are conserved in multi-cellular organisms as well. The best studied allele of error-prone transcription in yeast is the *rpb1*^*E1103G*^ allele, which raises the error rate of transcription 5-fold. Therefore, we used CRISPR/Cas9 technology to replicate this allele in *C. elegans* (*ama-1*^*E1120G*^) and monitored the fidelity of RNAPII. Because homozygous *ama-1*^*E1120G*^ worms are inviable, these experiments were performed on heterozygous animals, and these worms indeed displayed an increased G→A error rate in mRNA (5.4 × 10^−5^ ± 4.47 × 10^−7^), similar to yeast cells that carry the *rpb1*^*E1103G*^ allele (3D). No increase was observed in rRNA, indicating that this increase was not caused by unrelated cellular conditions that affected transcriptional fidelity (Fig. S8). We then wondered if the fidelity effect of this allele is also conserved in humans and used CRISPR/Cas9 technology combined with single cell cloning to generate multiple cell lines that carry an analogous mutation in HEK293 cells (*POLR2A*^*E1126G*^). Analysis of 2 independent homozygous *POLR2A*^*E1126G/E1126G*^ clones revealed that both cell lines display a ∼3-fold increase in transcription errors (6.85 × 10^−6^ ± 3.74 × 10^−7^/bp for clone 1 and 6.88 × 10^−6^ ± 1.51 × 10^−6^/bp for clone 2, Fig. 3E). This increase was primarily fueled by G→A errors (Fig. 3F), similar to the *rpb1*^*E1103G*^ allele in yeast and the *ama-1*^*E1120G*^ allele in worms. Taken together, these experiments demonstrate that the fidelity defects of yeast mutants can indeed be functionally conserved in multi-cellular organisms and highlight the threat of G→A errors to the fidelity of transcription across species. In addition, because the *POLR2A*^*E1126G/E1126G*^ cells display error-prone transcription, they provide a unique opportunity to understand how transcription errors affect human biology.

### Human patients with mutations in *POLR2A* that encode error-prone RNA polymerases

**Fig. 4:**
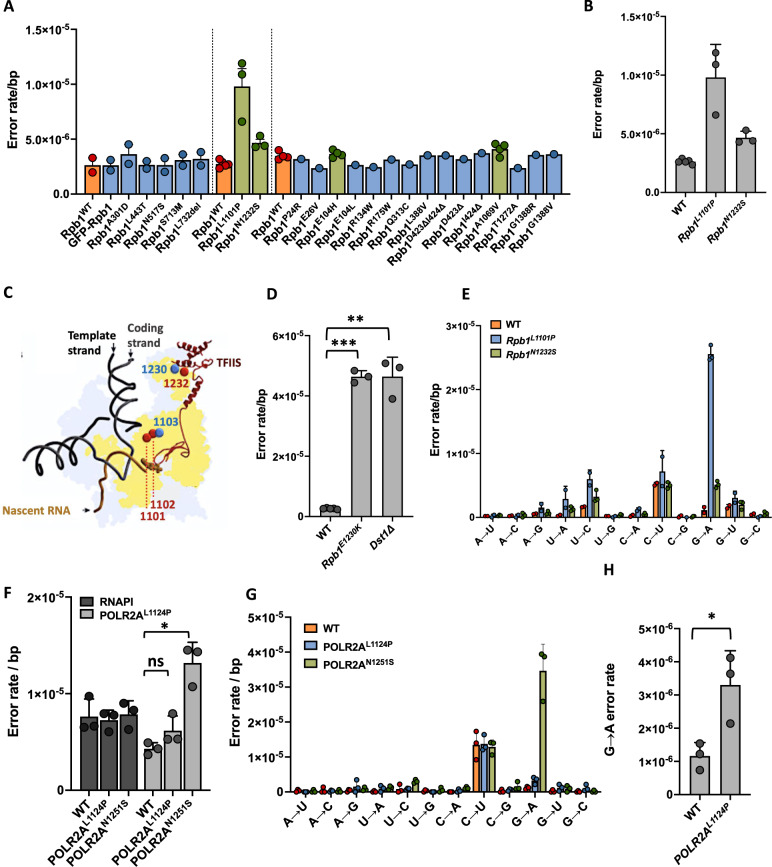
Increased error rates in yeast and human cells that carry patient-specific mutations. **A** Twenty-three patient-specific mutations were tested for their impact on transcriptional fidelity in yeast (*n* = 1). Four of these mutations (green bars) were selected for additional sequencing tests (*n* = 3). **B** During these additional tests, two of those mutations, *Rpb1*^*L1101P*^ (*P* = 0.0474) and *Rpb1*^*N1232S*^ (*P* = 0.0176) were found to display higher error rates compared to WT cells (*n* = 3). **C** The patient-specific mutations that were error-prone are located in close vicinity to alleles known to be error-prone, including the *Rpb1*^*E1103G*^ and *Rpb1*^*E1230K*^ allele. **D** Cells that carry the *Rpb1*^*E1230K*^ allele cannot couple TFIIS to Rpb1, mimicking a TFIIS knock out (*n* = 3, *P* = 0.0007 for *Rpb1*^*E1230K*^ and *P* = 0.0071 for *dst1Δ* cells*)* **E** The elevated error rate of *Rpb1*^*L1101P*^ and *Rpb1*^*N1232S*^ cells (*n* = 3) is primarily caused by an increased G→A error rate. **F** *POLR2A*^*N1251S*^ cells (*n* = 3, *P* = 0.0134) display an increased error rate of transcription that is primarily fueled by G→A errors (**G**). **H** Although the overall error rate of *POLR2A*^*N1124P*^ cells (*n* = 3) is not significantly different, these cells do display an increased G→A error rate (*P* = 0.0091), suggesting that *POLR2A*^*N1124P*^ proteins are indeed error-prone. In all cases, *n* is defined as biologically independent replicates. All experiments were analyzed by unpaired, two-tailed Welch’s *t*-tests using Prism software. *P < 0.05, **P < 0.01, ***P < 0.001. Error bars depict standard error from the mean. Source data are provided as a Source Data file.

Because the experiments described above demonstrate that human cells are capable of error-prone transcription, we wondered whether patients exist that express error-prone RNA polymerases. Interestingly, a primary^53^ and secondary^54^ cohort of patients was recently identified that carry mutations in POLR2A, the major catalytic subunit of RNAPII in human cells. These patients suffer from various symptoms, including muscle weakness, enlarged ventricles, white matter abnormalities and cerebellar problems. Several mutations identified in these patients cluster in regions that are essential for transcriptional fidelity, including the trigger loop, prompting us to perform an exploratory screen on a collection of yeast strains that carry analogous, patient-specific mutations in Rpb1, the yeast homolog of POLR2A^53^ (Fig. 4A). From this screen, 4 promising mutants were selected for further validation, 2 of which indeed displayed an elevated error rate (9.8 × 10^−6^ ± 2.82 × 10^−6^/bp for *rpb1*^*L1101P*^ cells and 4.67 × 10^−6^ ± 5.51 × 10^−7^/bp for *rpb1*^*N1232S*^ cells, Fig. 4B). One of these mutations affected amino acid 1101 of Rpb1 (POLR2A^L1124P^ in humans), which is only 2 amino acids upstream from the error-prone Rpb1^E1103G^ mutation in yeast and the error-prone POLR2A^E1126G^ mutation in humans (Fig. 4C). The second error-prone mutation (POLR2A^N1251S^ in humans) is located just downstream from amino acid 1230 in yeast. Interestingly, mutations at that location (rpb1^E1230K^) disconnect Rpb1 from the fidelity factor TFIIS^21,55^, which results in a 10-fold increase in transcription errors (4.64 × 10^−5^ ± 2 × 10^−6^/bp), mimicking cells in which TFIIS has been deleted (Fig. 4D). It is possible that a similar disconnect is responsible for the increased error rate of *rpb1*^*N1232S*^ cells. We further found that both alleles display an elevated G→A error rate (Fig. 4E), strengthening the relationship between the patient-specific alleles and the known, error-prone alleles that they flank.

Next, we wanted to confirm that these mutations result in error-prone transcription in human cells as well. To do so, we measured the error rate of transcription in genetically engineered HeLa cells that express either the *POLR2A*^*L1124P*^ or *POLR2A*^*N1251S*^ allele, or a WT *POLR2A* as a control. Excitingly, we found that cells that express the POLR2A^N1251S^ mutation indeed display an increased error rate fueled by G→A errors (1.32 × 10^−5^ ± 2.14 × 10^−6^/bp, Fig. 4E, F), similar to the *rpb1*^*N1232S*^ allele in yeast. Surprisingly though, the effect of the *POLR2A*^*L1124P*^ allele was modest compared to the analogous mutation in yeast (6.17 × 10^−6^ ± 1.52 × 10^−6^/bp), although *POLR2A*^*L1124P*^ cells did display an elevated G→A error rate (3.3 × 10^−6^ ± 1.03 × 10^−6^, Fig. 4F). It is possible though, that incomplete deactivation of the native POLR2A enzyme, or retention of the mutant POLR2A in the cytoplasm may have contributed to this observation (Fig. S9). Regardless, these experiments provide the first definitive proof that human patients exist that carry an error-prone RNA polymerase.

### Environmental causes of transcriptional mutagenesis

**Fig. 5:**
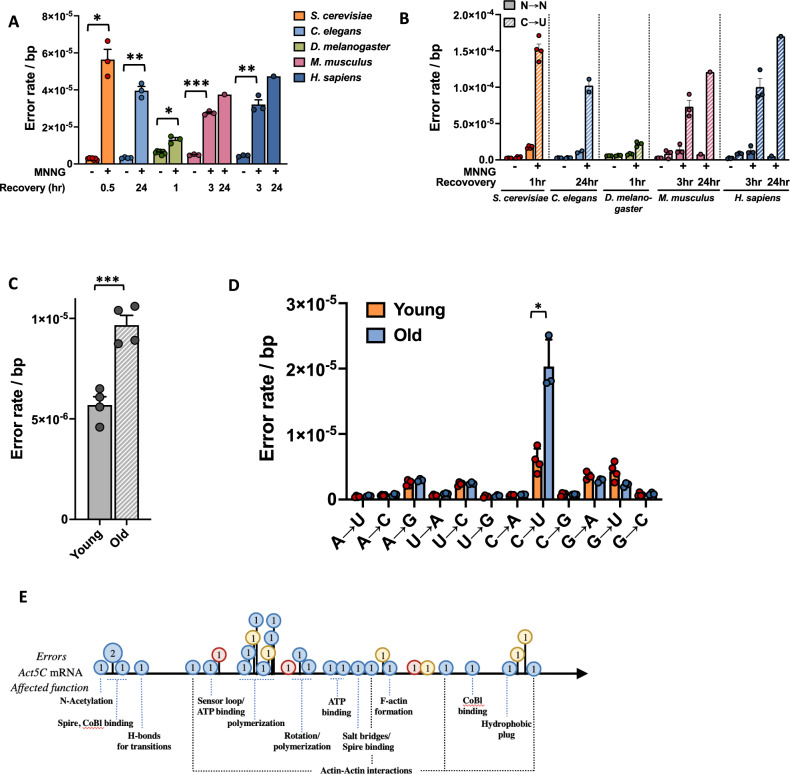
The error rate of transcription in multiple organisms of increasing complexity. **A** MNNG elevates the error rate of RNAPII in yeast (*n* = 7 for untreated cells and *n* = 3 for treated cells, *P* = 0.0104), worm (*n* = 4 for untreated worms and *n* = 3 for treated worms, *P* = 0.0036), fly (*n* = 7 for untreated flies and *n* = 3 for treated flies, *P* = 0.0193), mouse (*P* = 0.0002) and human cells (*P* = 0.0075) *n* = 3 for untreated cells, *n* = 3 for cells that were allowed to recover for a short time, and *n* = 1 for cells that recovered for a long time). **B** In each organism, the elevated error rate is primarily caused by a excessive C→U errors (n as in **A**). **C** Old flies display higher error rates than young flies (*n* = 4, *P* = 0.0009). **D** The increased error rate of old flies is primarily fueled by C→U errors (*n* = 4, *P* = 0.0162). **E** Age-related errors in flies can affect any aspect of protein structure and function, including actin molecules. Each circle indicates a base pair where errors were found. The number in the circle indicates the number of errors found at that base. Errors can result in non-synonymous amino acid changes (blue), synonymous changes (yellow) or premature stop codons (red). In all cases, n is defined as biologically independent replicates. All experiments were analyzed by unpaired, two-tailed Welch’s *t*-tests using Prism software. *P < 0.05, **P < 0.01, ***P < 0.001. Error bars depict standard error from the mean. Source data are provided as a Source Data file.

Multiple genes and protein structures that affect the fidelity of transcription in yeast are conserved in higher organisms. Accordingly, we wondered whether environmental factors that affect the error rate in yeast affect multi-cellular organisms as well. Currently, there are two environmental factors known to cause error-prone transcription in yeast: mutagen exposure^34^ (and the DNA damage it induces^28,30^) and natural aging^38^. One of the strongest transcriptional mutagens in yeast is the alkylating agent MNNG, which induces C→U errors through O^6^-methyl guanine adducts^35^. To determine if MNNG has the same effect on human cells, we exposed human fibroblasts for 1 h to 10μg/ml MNNG, replaced the medium and let the cells recuperate for 3 or 24 h to generate RNA molecules from damaged DNA templates and found that human cells display an ∼8-fold increase in transcription errors (3 × 10^−5^ ± 9.5 × 10^−6^/bp, Fig. 5A). Similar to yeast, this increase is primarily fueled by excess C→U errors, presumably due to O^6^-methyl guanine adducts that form on the DNA (Fig. 5B). These experiments demonstrate that exposure to mutagens can lead to enough DNA damage in human cells to elevate the error rate of transcription, and that this elevation can be maintained for an extended period of time after exposure. For two reasons, it is unlikely that these measurements were confounded by DNA mutations. First, our measurements were performed on cells that were grown into a non-dividing state by contact inhibition. Because the damaged cells were not actively replicating their genome and DNA replication is required to fix DNA damage into mutations, this quiescent state limits the mutagenic potential of MNNG adducts. Similarly, DNA replication is also required to fix ENU damage into mutations^56^ (ENU is a DNA alkylating agent as well). As a result, this feature can be exploited to bypass the impact of mutations on error measurements in the context of DNA damage^28,30,31^). Second, our measurements show that the error rate of transcription is >100-fold higher than the mutation rate in human cells^57,58^. For example, measurements in cultured human cells demonstrate that mutations occur at a frequency of 1.6 ± 1.2 × 10^−8^/bp and only increase several-fold in response to DNA damage^56–59^. Thus, it is unlikely that these experiments were confounded by DNA mutations introduced by MNNG exposure. Furthermore, because we observed a similar increase in C→U errors in worms, flies and mouse cells exposed to MNNG (Fig. 5A, B), we conclude that transcriptional mutagenesis is a universal consequence of exposure to MNNG.

Next, we sought to determine whether aging also affects the error rate of higher organisms. To do so, we compared the error rate of young flies (10 days) to aged flies (60 days) and found that old flies (9.66 × 10^−6^ ± 9.37 × 10^−7^/bp) indeed display higher error rates than young flies (5.69 × 10^−6^ ± 8.2 × 10^−7^/bp, Fig. 5c). Interestingly, this increased error rate was nearly entirely fueled by C→U errors (Fig. 5d), suggesting that the mechanism responsible for these errors is distinct from reduced proofreading or loss of fidelity factors. Instead, another molecular mechanism seems to be responsible. It is possible that one of these mechanisms is DNA damage, which is a hallmark of all aging organisms^60^. For example, lesions that specifically affect guanine bases, including the O^6^-methyl guanine lesions described above, could contribute to this age-related increase in C→U errors. Regardless of the mechanism, it is known that errors in mRNA transcripts cause protein misfolding^38^. Because misfolded proteins are part of the etiology of various age-related neurodegenerative diseases, including Alzheimer’s and Parkinson’s disease, an age-related increase in transcription errors could contribute to the progression of these diseases. Notably, a recent study indicated that silencing of MGMT, the human DNA repair protein that repairs O^6^-methyl guanine lesions, is a risk factor for Alzheimer’s disease in women and associated with a higher load of protein plaques and tangles^61^. Transcription errors could also affect aging organisms through other mechanisms though. For example, cytoskeletal structure is lost with age^62,63^, and errors in actin transcripts or other cytoskeletal components could contribute to this process by generating proteins that prevent additional subunits from being added to a growing chain (Fig. 5e).

### Human error rates, spectra and the genetic code

**Fig. 6:**
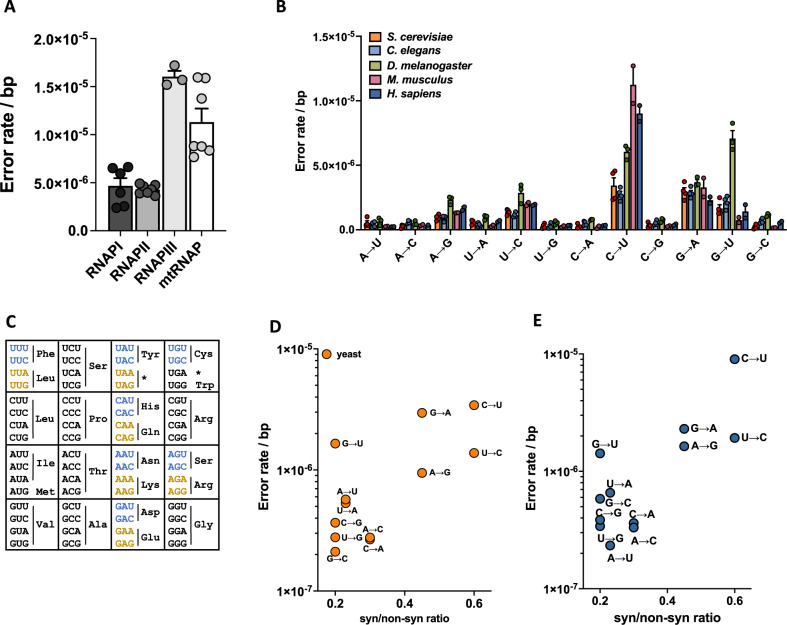
The error rate of transcription in various cell types and the genetic code. **A** The error rate of transcription in human cells (*n* = 7). **B** The error spectrum of transcription in organisms of increasing complexity (*n* = 7 for yeast, *n* = 4 for worms and flies, *n* = 3 for mice and *n* = 7 for human cells). **C** The genetic code is constructed in such a way that C→U and G→A errors do not result in mutated proteins if they occur in the “wobble” position. Errors that are more likely to result in a non-synonymous change, (as expressed by the ratio of synonymous to non-synonymous changes that can potentially result from that error), the lower the error rate is in yeast (**D**) and humans (**E**). Conversely, errors that are more likely to synonymous changes display higher error rates. In all cases, *n* is defined as biologically independent replicates. All experiments were analyzed by unpaired, two-tailed Welch’s *t*-tests using Prism software. *P < 0.05, **P < 0.01, ***P < 0.001. Error bars depict standard error from the mean. Source data are provided as a Source Data file.

Our measurements on untreated, primary human fibroblasts indicate that the error rate of human cells is 5.0 × 10^−6^ ± 1.43 × 10^−6^ for RNAPI, 4.7 × 10^−6^ ± 9.9 × 10^−8^ for RNAPII, 1.73 × 10^−5^ ± 4.53 × 10^−6^ for RNAPIII and 8.0 × 10^−6^ ± 4.81 × 10^−7^ for mtRNAP (Fig. 6A). These measurements provide the first reasonable estimate of the error rate of transcription in human cells, a useful benchmark for future experiments that examine the consequences of environmental exposure, genetic perturbations and human diseases on transcriptional mutagenesis. When we examined the error spectrum of RNAPII in human cells and compared it to error yeast, worms, flies and murine cells, we further noticed that in almost every case the error rate of RNAPII is primarily fueled by C→U and G→A errors (Fig. 6B). The similarities between these error spectra suggests that there might be an explanation for this phenomenon that transcends species.

One universal feature that is shared between species is the organization of the codon table. Intriguingly, the codon table is organized in such a way that amino acids encoded by two codons invariably have a C and a U, or a G and an A in the wobble position (Fig. 6C). As a result, the two most common transcription errors (C→U and G→A) do not result in a mutated protein if they affect the wobble base, suggesting that there is a beneficial relationship between the error rate, the error spectrum and the genetic code. To examine this relationship, we plotted the error rate of transcription against the likelihood that a transcription error will change the sequence of a protein. This analysis, which considers all three bases of each possible codon, demonstrates that transcription errors that are more likely to change a protein sequence or generate a stop codon (as expressed by a lower synonymous to non-synonymous ratio) occur less frequently than those that have a smaller chance of mutating an amino acid (Fig. 6D, E, Fig. S10). Thus, the error rate and spectrum of transcription is apparently biased to reduce the impact of errors on the proteome.

## Discussion

The fidelity of DNA replication, transcription and translation provide the foundation for life itself. As a result, multiple safety mechanisms have evolved to maintain the accuracy of these processes. For example, the fidelity of DNA replication is maintained by mechanisms that are both intrinsic and extrinsic to the replication machinery. One intrinsic mechanism is the proofreading capability of replicative DNA polymerases, which is built into the replication machinery itself and can correct mistakes that are made during DNA synthesis^1^. Polymerase proofreading is complemented by the mismatch repair machinery, an extrinsic safety mechanism that trails the replication fork and corrects mistakes that slip through this safety net^64^. At the time, the discovery of these safety nets provided deep insight into the mechanisms by which life propagates itself over countless generations and revealed the molecular underpinnings of various diseases that arise when these safety nets are removed^65,66^. Because transcription and translation occupy a similar position in the central dogma of life, similar multi-layered safety nets must have evolved to protect the fidelity of these processes. Conceivably, disruption of these mechanisms could contribute to human disease as well.

To identify these safety mechanisms, we used massively parallel sequencing technology to screen multiple model organisms for genes, alleles and environmental conditions that alter the fidelity of transcription. These experiments identified multiple factors that affect the fidelity of transcription. Interestingly, loss or mutation of multiple proteins raised the G→A error rate RNAPI and II, suggesting that these enzymes have a tendency to misincorporate adenine (rA) opposite cytosine (dC), and that multiple proteins are required to prevent these mistakes. This conclusion is also supported by in vitro experiments, which show that dC•rA mismatches are the most common mistakes made by isolated RNAPII on synthetic templates^12^. In addition, these experiments demonstrated that dC•rA mismatches are relatively efficiently extended^12^, and that RNAPII thus relies primarily on excision to prevent them. The increased G→A error rate of TFIIS and Rpb9 deficient cells is therefore most likely due to the role these proteins play in the excision of mismatched bases. Other mismatches, which occur at similar frequencies (including dC•rU and dA•rC mismatches)^12^ are more difficult to extend, which means that reduced excision only has a modest impact on their error rate (Fig S2). A similar rationale applies to Rpa12, Rpa34 and Rpa49, which enable the excision of mismatches by RNAPI^43^. Interestingly, it was previously shown that the error rate in *E. coli* (in particular for G→A errors) is relatively higher in rarely transcribed genes compared highly transcribed genes, raising the possibility that some transcripts benefit to a greater degree from fidelity factors than others^67^. Regardless, these observations illustrate how informative in vivo and in vitro datasets are when they are combined to study the fidelity of transcription. In vitro and in vivo datasets also complement each other in the context of the mitochondrial RNA polymerase. For example, it was previously shown that Rpo41 commits a large amount of A→G errors in vitro^68^, and we found that these errors are common in vivo as well (Fig. 2E). We also found that different mutations in Rpo41 result in different error spectra, suggesting that these mutations affect different safety mechanisms. Because Rpo41 does not contain proofreading activity, these mutations either affect nucleotide selection or extension. Our results show that the Rpo41^G1028A^ mutation causes a substantial increase in U→A errors, which are rarely made by Rpo41 in vitro. As a result, defects in mismatch extension are unlikely to raise the error rate of U→A substitutions; thus, it is more likely that nucleotide selection was affected by the Rpo41^G1028A^ mutation. In contrast, *rpo41*^*G1023A*^ cells display an increased rate of A→G and A→U errors, two errors that are already commonly made by Rpo41 in vitro. Accordingly, these errors would benefit the most from more efficient extension, suggesting that the Rpo41^G1023A^ mutation affects that aspect of Rpo41’s safety net.

When the molecular basis for the fidelity of DNA replication came into focus, it became possible to identify diseases that were associated with reduced fidelity. Accordingly, we wondered whether the in vivo experiments described above, in combination with the in vitro experiments that inspired them, could guide us towards diseases that are associated with reduced transcriptional fidelity. To do so, we identified two cohorts of patients that carry various mutations in *POLR2A* and found that one of these mutations indeed encodes a highly error-prone RNA polymerase. We anticipate though, that with the help of modern technology additional patients and diseases will be discovered in the future. For example, our results predict that mutations in the human homologs of TFIIS (TCEA1-3), Rpb9 (POLR2I), Rpa12 (POLR1H), Rpa34 (POLR1G), Rpa49 (POLR1E) and the mitochondrial RNA polymerase (POLRMT) could result in error-prone transcription. In addition, we found that O^6^-methyl guanine lesions cause transcription errors in human cells. Most likely then, patients that are deficient for MGMT^69,70^ (a DNA repair protein that repairs O^6^-methyl guanine lesions) display error-prone transcription as well. Our observations in yeast^38^ and flies further suggest that transcription errors are more common in aging individuals as well. The role of DNA damage, DNA repair and aging in transcriptional mutagenesis is especially interesting because transcription errors are known to result in protein misfolding, and can promote the aggregation of proteins associated with age-related diseases, including Alzheimer’s disease. Thus, DNA damage and aging could contribute to the progression of these diseases by elevating the error rate of transcription. In support of this idea, it was recently shown that the DNA repair protein MGMT is silenced in female AD patients with increased plaques and tangles^61^. To better understand the impact of errors on aging organisms (or those that have lost fidelity factors), it will be important to develop animal models that display error-prone transcription. For example, there are currently few or no models available to study the impact of rRNA and mtRNA errors on eukaryotic cells. To overcome this limitation, we identified 2 yeast strains that display error-prone transcription by RNAPI and 2 strains that display error-prone transcription by the mitochondrial RNAP. Together with *Rpa12Δ* cells, these cells could help researchers understand how rRNA and mtRNA errors affect cellular function. A similar strategy recently helped researchers understand how errors in mRNA affect the health of prokaryotic and eukaryotic cells. In these experiments, multiple error-prone cell lines were used to show that these errors can change cellular fate^71^, rewire metabolism^35^, generate mutant proteins^28–31^, induce protein aggregation^38^ and limit cellular lifespan^38^. It will now be important to translate these findings to multicellular organisms. To do so, we identified a strain of *C. elegans* that displays a 5-fold increase in the error rate of mRNA transcripts. We specifically searched for an error-prone strain of *C. elegans* because worms are genetically tractable organisms that can be rapidly screened by RNAi for modifiers of transcriptional mutagenesis, or genetically dissected to study the impact of transcription errors on organismal health. In addition, their translucent nature and short lifespan make them ideal tools for experiments for protein aggregation and aging experiments. Finally, there is an unmet need for human cells that display error-prone transcription, so that the impact of transcription errors on human biology can be dissected. To generate these cells, we used CRISPR/Cas9 technology to generate error-prone HEK293 cells that mimic the error-prone *rpb1*^*E1103G*^ allele in yeast. In addition, we characterized transgenic human cell lines that carry patient-specific mutations, which can be induced to express an error-prone RNA polymerase at will. We expect that these models will allow researchers to study the impact of transcription errors on key aspects of human biology.

Finally, our research demonstrates that C→U and G→A errors are the most common errors made across species. In part, this spectrum reflects the intrinsic error rate of RNAPII, as defined by the rate of incorporation, extension and excision of mismatched bases. For example, it was previously demonstrated that dC•rA mismatches are the most common mistakes made by RNAPII, which is likely to contribute to the G→A error rate in eukaryotic cells. Similar experiments showed that dG•rU mismatches are less frequently made, but relatively well extended, so that RNAPII has little time to excise these mismatches. Accordingly, the rapid extension of dG•rU mismatches could directly contribute to the C→U error rate in eukaryotic cells. A second mechanism that is likely to contribute to the spontaneous error rate is DNA damage. For example, O_6_-methyl guanine lesions are a powerful source of C→U errors, while cytosine deamination can contribute to the rate of G→A errors. Other lesions that could affect the fidelity of transcription are abasic sites, and various forms of oxidative damage, including 8-oxoguanine^28–31^. All these lesions have in common that they are relatively small and allow for efficient translesion synthesis, which facilitates transcriptional mutagenesis. Because RNAPII only makes one mistake every 250,000 bases on undamaged templates, but as many as one mistake every two bases on a damaged template, these lesions could have substantial impact on the error rate of transcription. One final possibility is that in some cases, cytosine deamination may also occur on RNA molecules. Such events could directly flip a cytosine base to uracil in mature transcripts and should thus be referred to as transcript errors rather than transcription errors.

One intriguing aspect that is directly related to the error spectrum of transcription, is that the codon table is organized in such a way that amino acids invariably have a C and a U, or a G and an A in the wobble position (Fig. 6C). As a result, C→U and G→A errors do not result in mutant proteins if they occur in the wobble position, indicating that the error rate of transcription is biased to limit the impact of transcription errors on the proteome (Fig. 6D, E). There are two potential explanations for this observation. In our opinion, the most likely explanation is that the error spectrum evolved in response to the organization of the genetic code. If C→U and G→A errors are less consequential than other errors, there is less selective pressure to prevent them. A second, more provocative explanation is that this evolutionary relationship is reciprocal, and that the genetic code evolved in concert with the error rate of transcription. Although many explanations have been offered for the organization of the genetic code, one interesting hypothesis is that the genetic code evolved to limit the impact of genetic mutations and translation errors on the proteome^72–76^. Since our data shows that the genetic code is also biased to limit the impact of transcription errors on the proteome, it may now be possible to add transcription errors to this hypothesis, raising the possibility that the error rates of DNA replication, transcription and translation together contributed to the evolution of the genetic code.

## Methods

All experiments performed in this study comply with relevant ethical regulations as stipulated by the IACUC committee at the University of Southern California.

### Library construction and sequencing

Library preparation 1100 ng of enriched mRNA was fragmented with the NEBNext RNase III RNA Fragmentation Module (E6146S) for 25 min at 37 °C. RNA fragments were then purified with an Oligo Clean & Concentrator kit (D4061) by Zymo Research according to the manufacturer’s recommendations, except that the columns were washed twice instead of once. The fragmented RNA was then circularized with RNA ligase 1 in 20 µl reactions (NEB, M0204S) for 2 h at 25 °C after which the circularized RNA was purified with the Oligo Clean & Concentrator kit (D4061) by Zymo Research. The circular RNA templates were then reverse transcribed in a rolling-circle reaction by first incubating the RNA with for 10 min at 25 °C to allow the random hexamers used for priming to bind to the templates. Then, the reaction was shifted to 42 °C for 20 min to allow for primer extension and cDNA synthesis. Second strand synthesis and the remaining steps for library preparation were then performed with the NEBNext Ultra RNA Library Prep Kit for Illumina (E7530L) and the NEBNext Multiplex Oligos for Illumina (E7335S, E7500S) according to the manufacturer’s protocols. Briefly, cDNA templates were purified with the Oligo Clean & Concentrator kit (D4061) by Zymo Research and incubated with the second strand synthesis kit from NEB (E6111S). Double-stranded DNA was then entered into the end-repair module of RNA Library Prep Kit for Illumina from NEB, and size selected for 500–700 bp inserts using AMPure XP beads. These molecules were then amplified with Q5 PCR enzyme using 11 cycles of PCR, using a two-step protocol with 65 °C primer annealing and extension and 95 °C melting steps. Each library was normalized and sequenced on Illumina’s NovaSeq 6000, using the NovaSeq SP flow cell v1.5 chemistry 500 cycles kit at 250 bps paired-end sequencing (Illumina, San Diego, CA). Raw sequencing data was converted to industry standard Fastq files using BCL2FASTQv1.8.4.

### Error identification

We have developed a robust bioinformatics pipeline to analyze circ-seq datasets and identify transcription errors with high sensitivity^35,40^. First, tandem repeats are identified within each read (minimum repeat size: 30 nt, minimum identity between repeats: 90%), and a consensus sequence of the repeat unit is built. Next, the position that corresponds to the 5′ end of the RNA template is identified (the RT reaction is randomly primed, so cDNA copies can “start” anywhere on the template) by searching for the longest continuous mapping region. The consensus sequence is then reorganized to start from the 5′ end of the original RNA fragment, mapped against the genome with tophat (version 2.1.0 with bowtie 2.1.0) and all non-perfect hits go through a refining algorithm to search for the location of the 5′ end before being mapped again. Finally, every mapped nucleotide is inspected and must pass **5** checks to be retained: (**1**) it must be part of at least 3 repeats generated from the original RNA template; (**2**) all repeats must make the same base call; (**3**) the sum of all qualities scores of this base must be >100; (**4**) it must be >2 nucleotides away from both ends of the consensus sequence; (**5**) each base must be covered by ≥200 reads with <1% of these reads supporting a base call different from the reference genome. This final step filters out polymorphic sites and potential RNA-editing events. For example, if a base call is different from the reference genome, but is present in 100 out of 200 reads, it is not labeled as an error but as a heterozygous mutation. A similar rationale applies to low-level mutations and RNA editing events. Each read containing ≥1 mismatch is filtered through a second refining and mapping algorithm to ensure that errors in calling the position of the 5′ end cannot contribute to false positives. The error rate is then calculated as the number of mismatches divided by the total number of bases that passed all quality thresholds.

### Yeast culture

Yeast Culture and Treatments Single colonies were inoculated in YAPD and incubated overnight at 30 °C in a rotating wheel. In the morning, the optical density (OD600 nm) of each culture was measured using Thermo Scientific’s Nanodrop 2000C and cells were re-inoculated at an OD600 0.05–0.1 in 50 ml YAPD flasks and incubated in an orbital shaker at 30 °C. Cells were then grown to an OD of 0.25–0.5 and arrested with 50 µg/ml alpha-mating factor. After 2.5 h, the cells were visualized under a microscope to confirm they were arrested, and then treated, or not, for 40 min with MNNG (2.5–20 µg/ml). After treatment, the cells were washed 3 times in PBS to remove mutagenic compounds and RNA was extracted with the RiboPure Yeast kit from Ambion Technologies (PN1926M) according to the manufacturer’s protocol, with the exception that the RNA was never exposed to temperatures higher than 65 °C. After isolation of total RNA, we purified mRNA with either one or two rounds of poly(A) purification using the Sigma GenElute mRNA miniprep kit (MRN70-1KT) and constructed circle-seq libraries.

### Construction of mutant yeast strains

The RPO41 gene was amplified from WT BY4741 yeast cells with the high-fidelity DNA polymerase Q5 from NEB, inserted into a pRS316 integration vector with a URA3 auxotrophy marker and the desired mutations were inserted into the RPO41 gene with the QuickChange site-directed mutagenesis kit from Agilent (#200523). Mutated plasmids were then transformed into WT yeast cells with standard lithium acetate protocols, and grown on -URA medium to select for transformants. Individual clones were then isolated and the RPO41 locus was sequenced to identify at least 3 positive transformants.

### Mammalian cell culture, treatment and RNA extraction

Primary cells derived from the ears of 3-month-old mice, or primary cells from a 42-year-old, clinically healthy female (Coriell institute GM10901) were grown in 5% oxygen tension in standard DMEM medium, containing high glucose, high pyruvate, 10% FBS, and 1% Pen-Strep. Once the cells reached confluence, they were maintained at that state for 3 additional days to stop cell division. Cells were then exposed to 50 µM MNNG for 1 h and washed twice to remove MNNG particles. To isolate RNA, 1 ml of Trizol was added to each plate, cells were scraped off, and collected in a 1.5 ml Eppendorf tube. The cells were then incubated in Trizol for 5 min, after which 200 µl of chloroform was added to the mixture followed by a second incubation for 5 min. After that, the cells were spun down at 4 °C for 10 min at 10,000 g and 530 µl of the aqueous phase was aspirated and added to the RNA binding/ethanol solution of the RiboPure Yeast kit from Ambion Technologies (PN1926M). After isolation of total RNA, we purified mRNA with either one or two rounds of poly(A) purification using the Sigma GenElute mRNA miniprep kit (MRN70-1KT) and constructed circle-seq libraries. HEK293 cells were purchased from ATCC (CRL-1573).

### Culture of HeLa cells that contain patient specific mutations

The open reading frame of human POLR2A was modified to contain a B10 epitope, EGFP, six His residues and mutated to replace Asp 792 by As, resulting in resistance to α-amanitin. Then, POLR2A point mutations were introduced through the Quickchange protocol (Stratagene) and verified by DNA sequencing. Stable doxycycline inducible cell lines were then transfected with pCDNA5/FRQT/TO and pOG44 into HeLa FRT cells carrying the TET repressor and placed on antibiotic selection. HeLa cells grown in 5% oxygen tension in standard DMEM medium, containing high glucose, high pyruvate, 10% FBS, and 1% Pen-Strep. The expression of GFP-tagged POLR2A was induced by treatment with 1 μg/mL doxycycline for 48 h and treated with 2.5 μg/mL α-aminitin for 36 h to degrade endogenous POLR2A proteins. To isolate RNA, 1 ml of Trizol was added to each plate, cells were scraped off, and collected in a 1.5 ml Eppendorf tube. The cells were then incubated in Trizol for 5 min, after which 200 µl of chloroform was added to the mixture followed by a second incubation for 5 min. After that, the cells were spun down at 4 °C for 10 min at 10,000 g and 530 µl of the aqueous phase was aspirated and added to the RNA binding/ethanol solution of the RiboPure Yeast kit from Ambion Technologies (PN1926M). After isolation of total RNA, we purified mRNA with either one or two rounds of poly(A) purification using the Sigma GenElute mRNA miniprep kit (MRN70-1KT) and constructed circle-seq libraries.

### Worm treatment and RNA extraction

Wild-type N2 Bristol strains were maintained on plates with OP50 at 20 °C. For the treatment, worms were washed off the plates and incubated with 9 mM MNNG in S basal (or M9) buffer for 60 min at 100 rpm in a 20 °C incubator. The worms were collected by centrifugation at 800 × *g* for 5 min and washed with fresh buffer three times. Worms were then lysed with the RiboPure Yeast kit from Ambion Technologies (PN1926M) by drowning them in the lysis medium and phenol chloroform, and bead-based disruption was used to open up the cells according to the manufacturer’s protocol. After isolation of total RNA, we purified mRNA with either one or two rounds of poly(A) purification using the Sigma GenElute mRNA miniprep kit (MRN70-1KT) according to the manufacturer’s protocol, with the exception that the RNA was never exposed to temperatures above 65 °C for longer than 3 min.

### Fly treatment and RNA extraction

Wild-type Canton-S strains were maintained in vials containing standard dextrose-based medium. For aging experiments, at least 30 male flies were aged per biological replicate until they were 60 days old. Vials were flipped every 2 days. For treatment, flies were starved for 3 h prior to exposure and then flipped into vials that contained a bed of kim-wipes saturated with dextrose-infused water and 64 mM MNNG for 6 h. Ingestion of MNNG was confirmed during preliminary experiments in which dextrose infused water was colored with a blue dye, so that it could be visualized under a stereoscope in the intestines of the flies. Flies were then lysed with the RiboPure Yeast kit from Ambion Technologies (PN1926M) by drowning them in the lysis medium and phenol chloroform, rapid homogenization with a pestle, and bead-based disruption according to the manufacturer’s protocol. After isolation of total RNA, we purified mRNA with either one or two rounds of poly(A) purification using the Sigma GenElute mRNA miniprep kit (MRN70-1KT) according to the manufacturer’s protocol, with the exception that the RNA was never exposed to temperatures above 65 °C for longer than 3 min.

### Introduction of POLR2A mutation in HEK-293 cells

Guide RNA sequences targeting POLR2A (gRNA A: TTGATGAGCTCCTTAAGTCG; gRNA B: TCGCTTACTGTCTTCCTGTT) were designed using the http://crispr.mit.edu software tool (Cambridge, MA). Guide RNAs were cloned in the humanized S. pyogenes Cas9-D10A vector pX335 (Addgene plasmid 42335. Green fluorescent protein (GFP) expressed from the pmaxGFP plasmid (Lonza) was used as a screening tool. Plasmids were isolated using QIAGEN Plasmid Midi Kit. A 178-base single-stranded donor oligonucleotide for introduction of POLR2A E1126G mutation (GA*A*C*CTGCCACCCAGATGACCTTGAATACCTTCCACTATGCTGGTGTGTCTGCCAAGAATGTGACGCTGGGTGTGCCCCGACTTAAA1GGG2CTCATCAACATTTCCAAGAAGCCAAAGACTCCTTCGCTTACTGTCTTCCTGTTGGGCCAGTCCGCTCGAGATGCTGAGAGAGCCA*A*G*G; A1 was generated as an ultramer by Integrated DNA Technologies. * indicates phosphothiorate linkage to prevent degradation by exonucleases.

Human embryonic kidney (HEK)−293 cells were purchased from the American Type Culture Collection (Manassas, VA) and cultured according to the supplier’s instructions. A total of 4.94 μg gRNA-Cas9n plasmids, 50 ng pmaxGFP plasmid, and 100 pmol single-stranded oligonucleotide were introduced to HEK-293 cells (2 × 106) by nucleofection using Amaxa’s Nucleofector device (Lonza), the Cell Line Nucleofector Kit V (Lonza), and Program Q-001. Subcloning was performed to select correctly targeted clones. Briefly, two days after nucleofection, GFP positive, viable single cells were sorted into fifty 96-well plates using MoFlo Astrios EQ cell sorter (Beckman Coulter). When clones were passaged, some HEK-293 cells were collected and genomic DNA was isolated using QuickExtract DNA Extraction Solution (Lucigen, Middleton, WI). To identify clones carrying POLR2A E1126G mutation, a 323 bp fragment of POLR2A was amplified by PCR using POLR2A specific primers in 138 clones and cut with restriction enzyme AflII, and candidate clones were subjected to Sanger sequencing analysis. POLR2A mutant clones were identified by sequence comparison to wild-type HEK-293 cells. Culturing conditions included growth in 5% oxygen tension in standard DMEM medium, containing high glucose, high pyruvate, 10% FBS, and 1% Pen-Strep.

### Generation of mutant *C. elegans* strains

Purified Cas9 protein was reconstituted with 2 gRNAs ((ACTTGGAGTGCCGAGATTGA and GTCAAGAATACAGTCAACGA) and 1 oligo product (TCGGCGAAGAATGTGACACTTGGAGTGCCGAGAttaaaaggaatcataaacgttagcaaaaccttgaaaacaccatcaTTGACTGTATTCTTGACGGGAGCGGCTGCCAAGGA) that were directly injected into the gonad of the recipient N2-worms. A dpy-10 sgRNA was added to this DNA mix to create a co-CRISPR dominant phenotype and mark which injections were repair competent. F1 progeny were first screened for the dominant co-CRISPR phenotype and then 100 F1 progeny from plates with multiple that displayed the dominant marker were singled and screened for gene insertion by PCR and restriction digest to detect heterozygosity for the desired *ama-1*^*E1120G*^ mutation. Since homozygous *ama-1*^*E1120G*^ worms were inviable, heterozygous worms were maintained opposite a homozygous lethal balancer chromosome (nT1) on plates that were spotted with OP50 and held at 20 °C.

### Biological materials

All biological materials are available upon request to M.V. or Dr. Jeffrey Strathern.

### Statistics and reproducibility

Experiments over several years have demonstrated that the circle-sequencing protocol is highly reproducible, with measurements of biologically identical samples reporting the same error frequencies and rates over the course of 6 years, most likely due to the large numbers of errors detected. Based on these empirical observations, we designed our experiments with an *n* of 3 biologically independent replicates in mind. However, multiple experiments contained additional biological replicates of untreated or WT samples, which served to increase the size of our total replicate pool, and as a standard to ensure accuracy of our assays. In each case, these additional samples yielded highly similar results that were indistinguishable from previous replicates. In addition, multiple samples were re-sequenced in order to acquire more data, improve statistical power or fleshing out error spectra. These resequencing efforts were always indistinguishable from initial measurements. The experiments were not randomized. However, all experiments were performed at the bench by multiple investigators. The circle-sequencing protocol takes 16–24 h to complete over a 2-day timespan and requires multiple researchers to process various stages. During these stages, samples are labeled by numbers and divided over 2 or three researchers, each of which receives one or two of the replicates for a certain condition or genotype, so that all replicates are the result of at least 2 pairs of hands. In addition, no efforts were made to control the order in which the samples were processed. Although the investigators were not technically blinded to allocation during experiments and outcome assessment, all experiments were performed at USC and UPenn, and analyses of sequencing data from these experiments were performed by a second, remote team located in Mississippi State. Statistical analyses were performed using standard two tailed, unpaired assuming no identical standard deviation t-tests with Prism Graphpad software with the help of the bio-informatics and statistics core at USC. Various other statistical tests were performed as well though, which yielded qualitatively identical results.

### Reporting summary

Further information on research design is available in the Nature Portfolio Reporting Summary linked to this article.

## Supplementary information

Supplementary Info

Reporting Summary

## Data Availability

All the sequencing data generated from yeast, worms, flies, mice and human cells and any accession codes will be shared on the Sequence Read Archive (SRA), the primary NIH-funded archive for high throughput datasets. The sequencing data generated in this study have been deposited in the SRA database (https://www.ncbi.nlm.nih.gov/sra) under accession codes: PRJNA924347, PRJNA923732, PRJNA923308, PRJNA922590, PRJNA672117, PRJNA673738, PRJNA673853, PRJNA673744. Source data are provided with this paper.

## Source data

Source data file

## Code availability

Code to analyze circ-seq datasets is available at https://github.com/jfgout/circseq-seqan2. This code can also be accessed through Zenodo: 10.5281/zenodo.7591325^77^.
