# Supplementary material for: Evolutionary conservation of the fidelity of transcription
Source: Nat Commun. 2023 Mar 20;14:1547. doi: 10.1038/s41467-023-36525-w (PMC10027832; doi:10.1038/s41467-023-36525-w)
Supplement: Supplementary file 1 — Supplementary Info [file 41467_2023_36525_MOESM1_ESM.pdf]

Figure S1

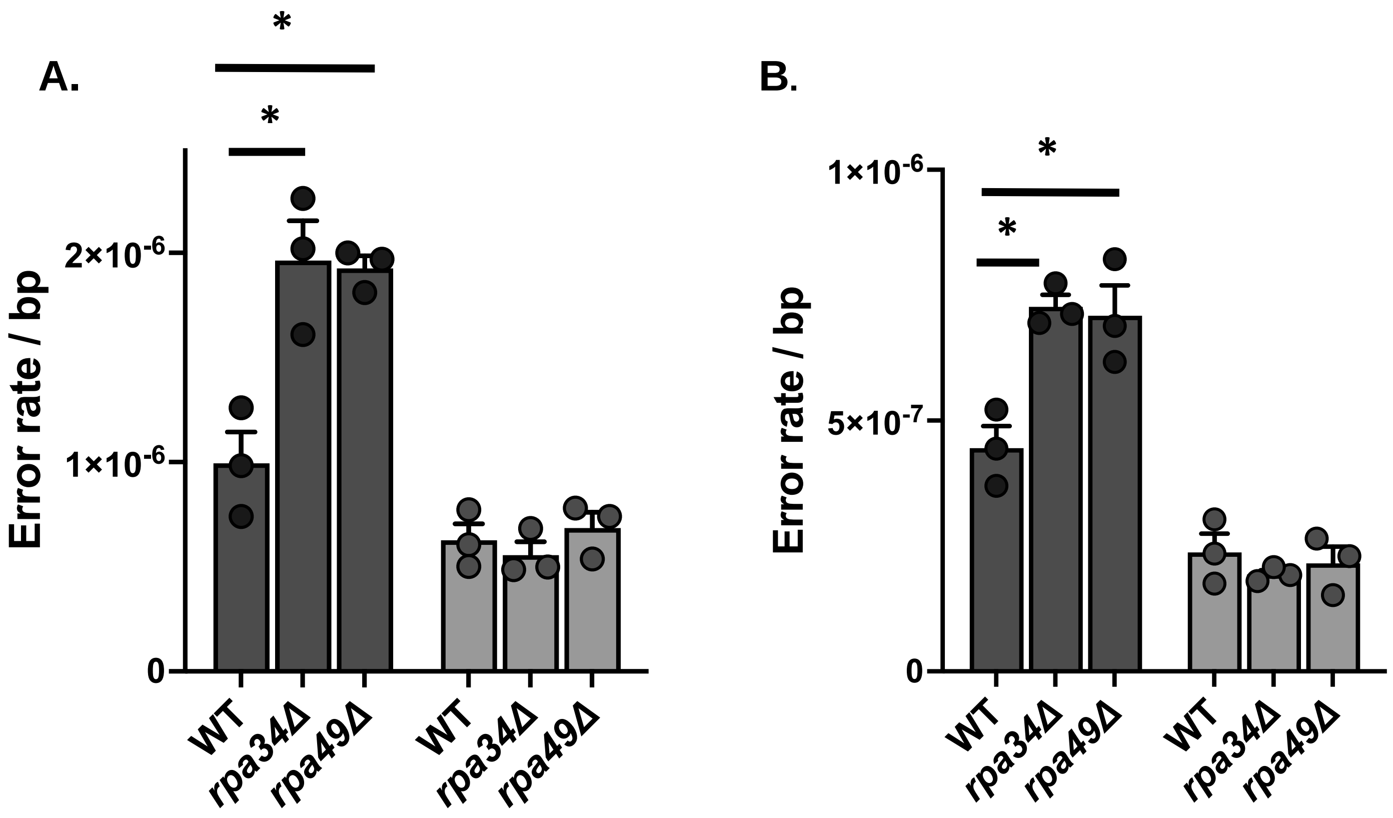

Figure S1 | The error rate of RNAPI mutants in yeast. A-B. Loss of Rpa34 (P = 0.0178) and Rpa49 (P = 0.0151) elevates the insertion (**A**) and deletion rate (**B,** P = 0.0102 of *rpa34Δ* cells and P = 0.0275 for *rpa49Δ* cells) of RNAPI in yeast. N=3 biologically independent replicates for all samples. All experiments were analyzed by unpaired, 2-tailed Welch's t-tests using Prism software. * = P<0.05, ** = P<0.01, *** = P <0.001. Error bars depict standard error from the mean. Source data are provided as a Source Data file.

Figure S2

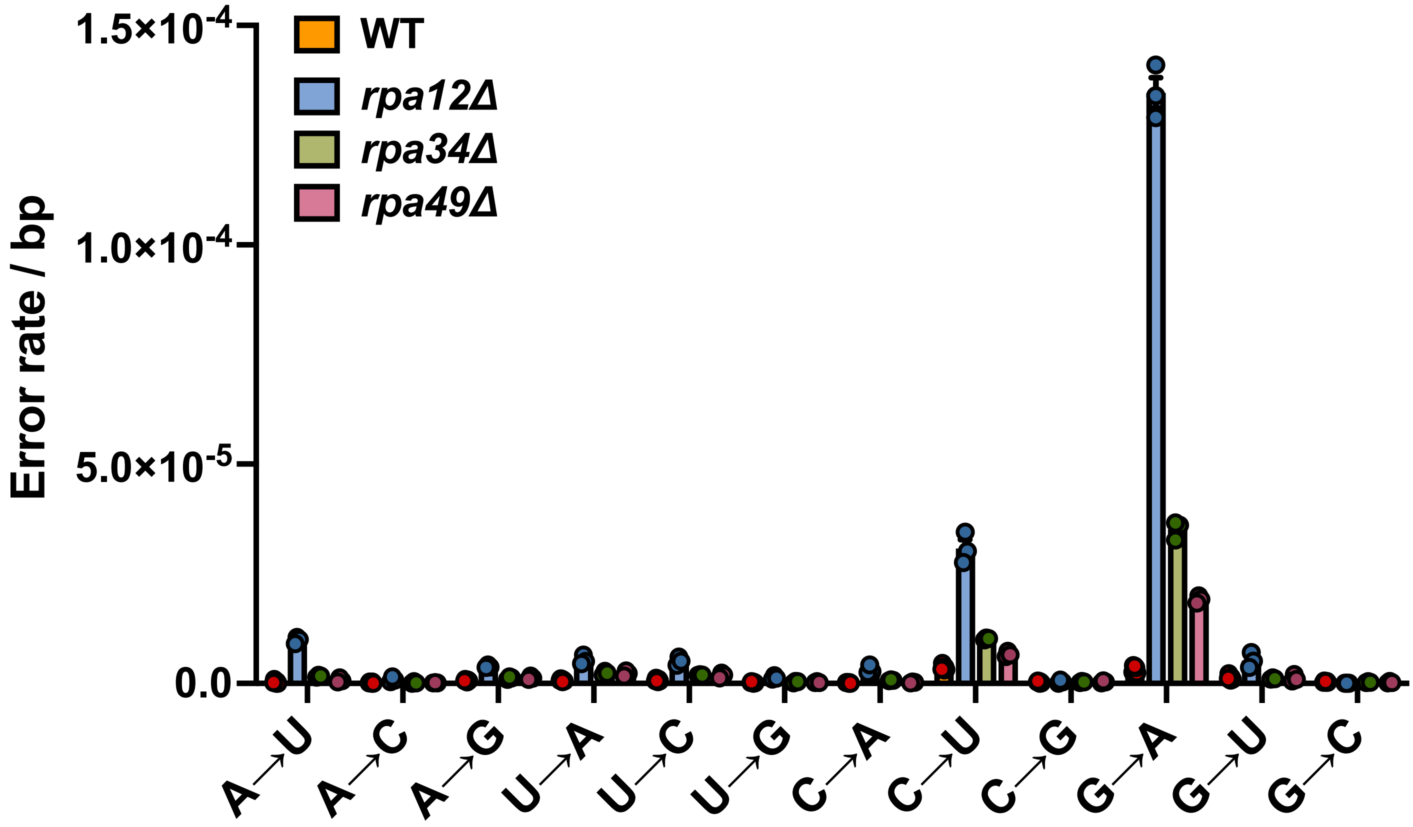

Figure S2 | The error spectrum of RNAPI in WT cells and cells that carry an error-prone version of RNAPI. Error bars depict standard error from the mean. N=7 biologically independent replicates for WT cells and n=3 for mutant strains. Source data are provided as a Source Data file.

Figure S3

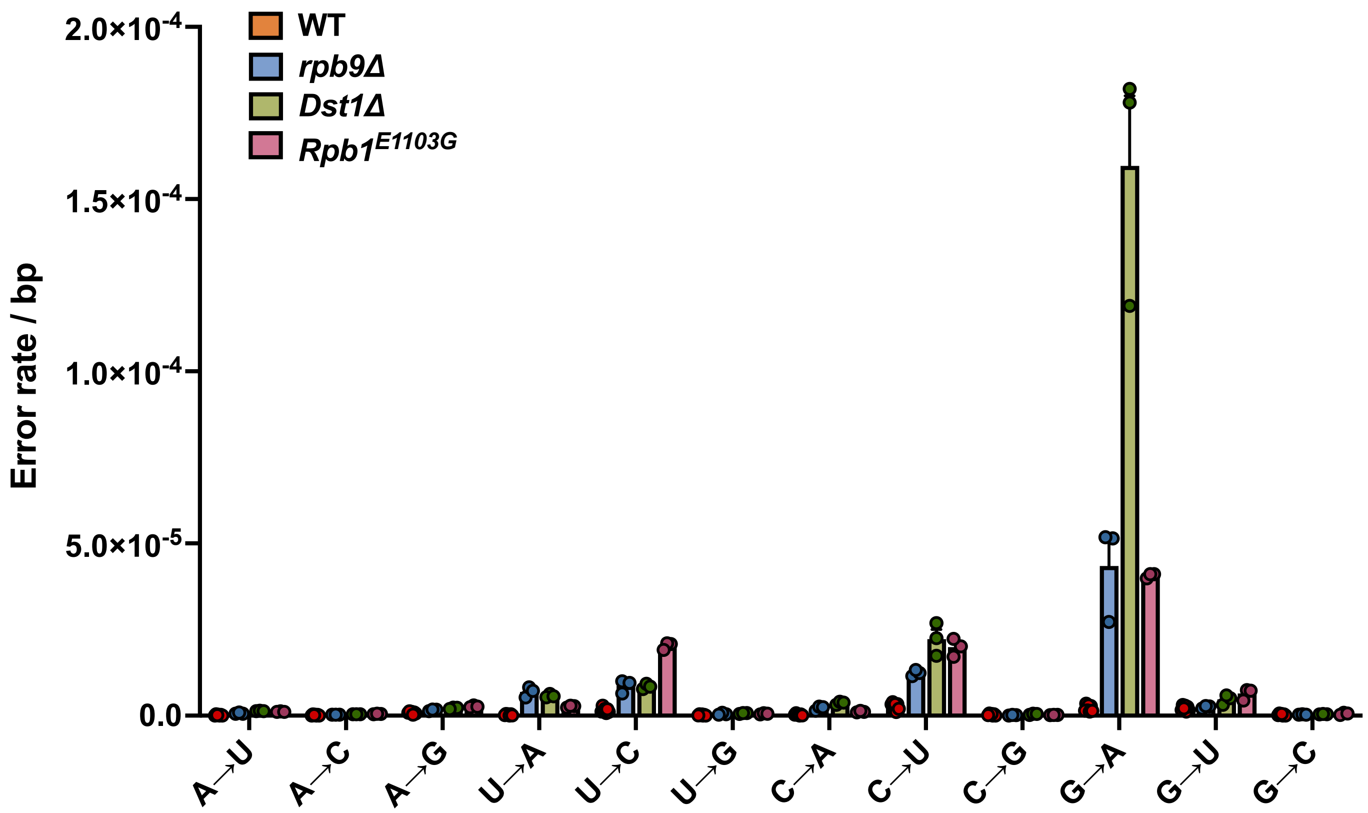

Figure S3 | The error spectrum of RNAPII in WT cells and cells that carry an error-prone version of RNAPII. Error bars depict standard error from the mean. N=7 biologically independent replicates for WT cells and n=3 for mutant strains. Source data are provided as a Source Data file.

Figure S4

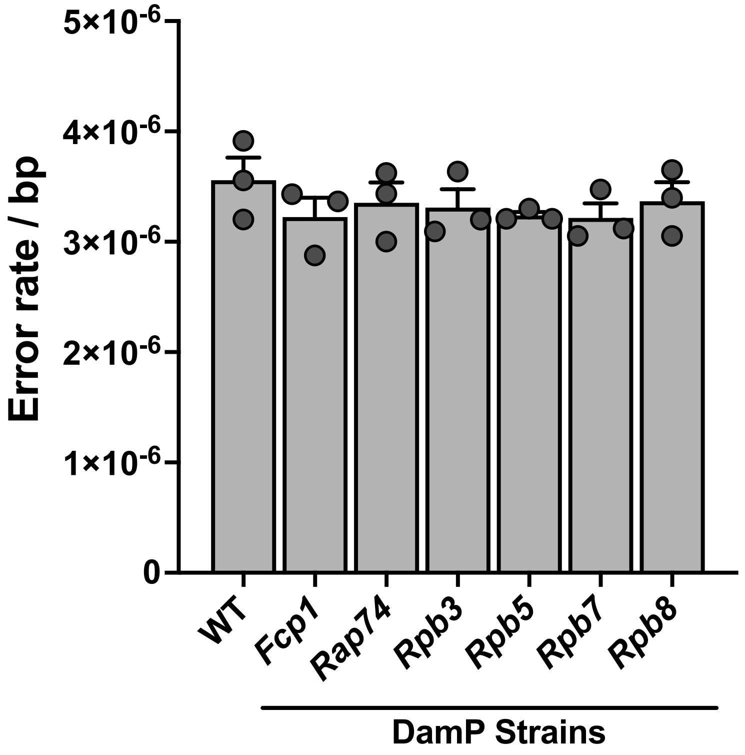

Figure S4 | Knock-down of various essential RNAPII subunits does not affect the error rate of transcription. Error bars depict standard error from the mean. N=3 biologically independent replicates for all strains. Source data are provided as a Source Data file.

Figure S5

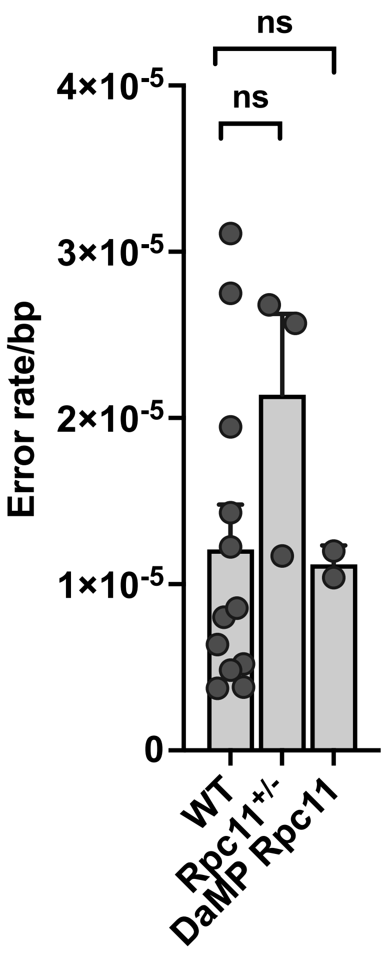

Figure S5 | Heterozygous Rpc11$^{+/-}$ cells do not display an increased error rate. All experiments were analyzed by unpaired, 2-tailed Welch's t-tests using Prism software. Error bars depict standard error from the mean. N=12 biologically independent replicates for WT cells and n=3 for mutant cells. Source data are provided as a Source Data file.

Figure S6

A

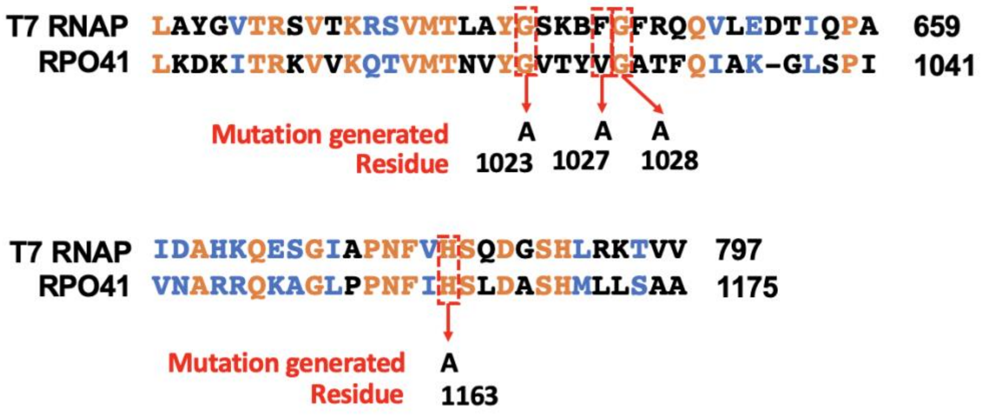

B

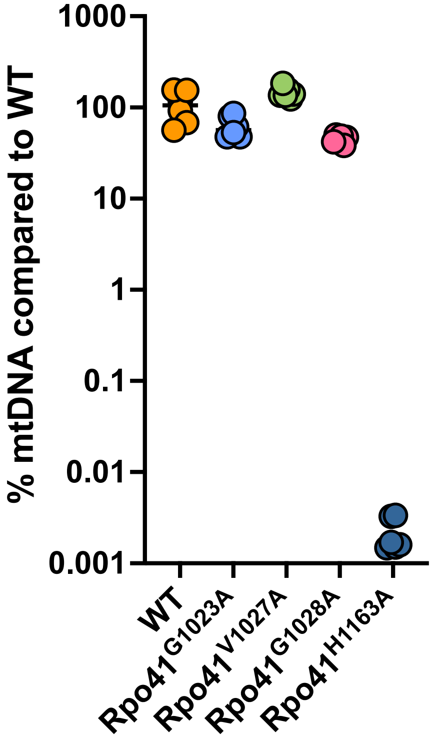

Figure S6 | A. Protein alignment of the T7 RNAP and the yeast mitochondrial RNAP Rpo41. Four mutations were identified in the T7 RNAP that result in error-prone transcription (red boxes). We generated identical mutations in the Rpo41 protein (Rpo41^{G1023A}, Rpo41^{V1027A}, Rpo41^{G1028A} and Rpo41^{G1163A}) by genetic substitution of the endogenous *Rpo41* gene. Identical amino acids are

depicted in orange and highly similar amino acids in blue. **B. mtDNA levels of WT and mutant strains of Rpo41.** The H1163A strain displays a large decrease in mtDNA copynumber. N= 6 biologically independent replicates for all strains. Source data are provided as a Source Data file.

Figure S7

```
Yeast TFIIS      ---MDSK-EVLVHVKNLEKNKSNDA-AVLEILHVLDKEFVPTEKLLRETKVGVEVNKFKK   55
Worm T24H10.1    MSALEETQSLCKQVDDICNNGMESVEQCNKLLDQLSK-IPMSIEIIQKTNIGIKVNMMRK   59

Yeast TFIIS      S-TNVEISKLVKKMISSWKDAINKNKRSRQAQQHHQDHAPGNAEDKTTVGESVN---GV-   110
Worm T24H10.1    KVTDDAVAKRAKNIIKDWKNVVDGKSKSQDDGG----APPAKKHRKESVEEAKPEKKKIE   115

Yeast TFIIS      ---QQPAS-SQSDAMKQDKYVSTKPRNSKNDGVDTAIYHHKLRDQVLKALYDVLAKESEH   166
Worm T24H10.1    APYKRPEPSSRPEIVAQFASASFPPKHLENDETRLK--S---AQLLLSAL------RFGD   164

Yeast TFIIS      PPQSILHTA---KAIESEMNKVNNCDTNEAAYKARYRIIYSNVISKNNPDLKHKIANGDI   223
Worm T24H10.1    MPQGTLDPEELAVQIEEKLYSVH--RDTNKSYSAAVRSRIFNLRDKKNLALRENVLTGVV   222

Yeast TFIIS      TPEFLATCDAKDLAPAPLKQKIEEIAKQNLYNAQGATIERSVTDRFTCGKCKEKKVSYYQ   283
Worm T24H10.1    RAEKFATMTSEEMASAEIREMRDKFTKEAILEHQMSVQQGTPSDMFKCGKCGKKNCTYTQ   282

Yeast TFIIS      LQTRSADEPLTTFCTCEACGNRWKFS   309
Worm T24H10.1    LQTRSSDEPMTTFVFCLECGNRWKFC   308
```

Figure S7 | Protein alignment of the *S. cerevisiae* TFIIS protein and the *C. elegans* T24H10.1 protein. TFIIS and T24H10.1 are 27% identical (orange amino acids) and 54% highly similar (blue amino acids). Highly similar amino acids are those that share strongly similar properties, roughly equivalent to scoring > 0.5 in the Gonnet PAM 250 matrix, such as: STA, NEQK and MILV. More detailed descriptions can be found on the Clustal Omega Alignment tool website and FAQs.

Figure S8

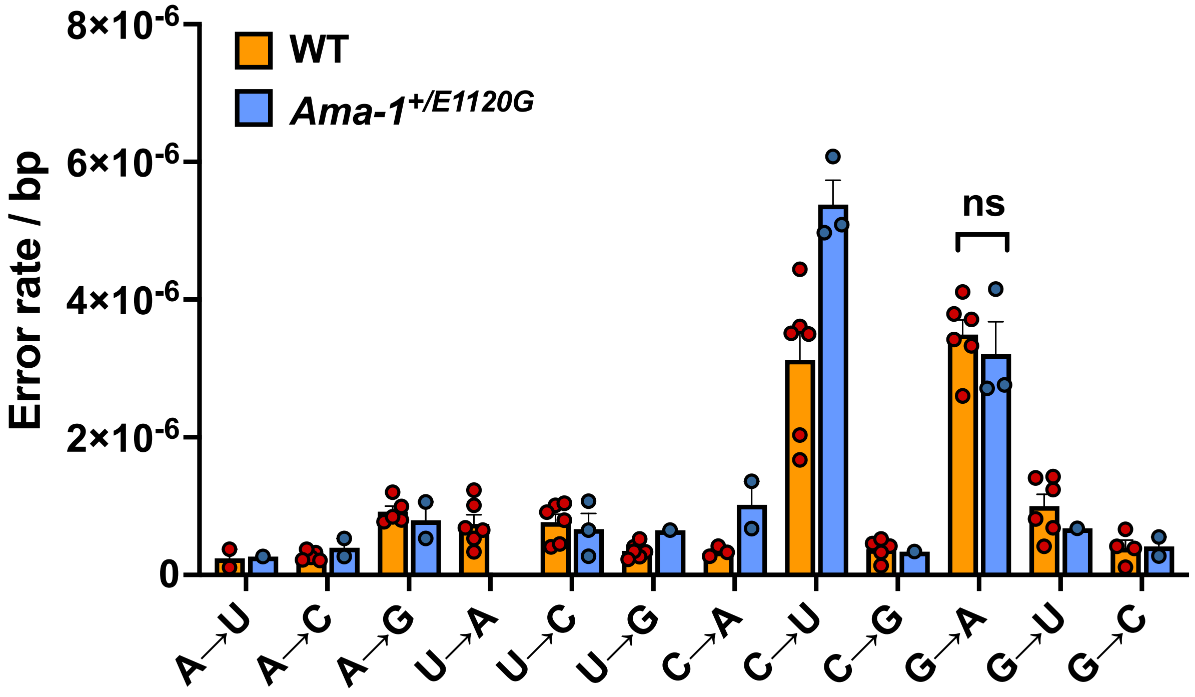

Figure S8 | Error spectrum of RNAPI in WT and mutant Ama-1$^{+/E1120G}$ worms. In contrast to RNAPII, RNAPI does not display an increase in G to A errors in Ama-1$^{+/E1120G}$ worms. All experiments were analyzed by unpaired, 2-tailed Welch's t-tests using Prism software. Error bars depict standard error from the mean. N=6 biologically independent replicates for WT cells and n=3 for mutant cells. Source data are provided as a Source Data file.

Figure S9

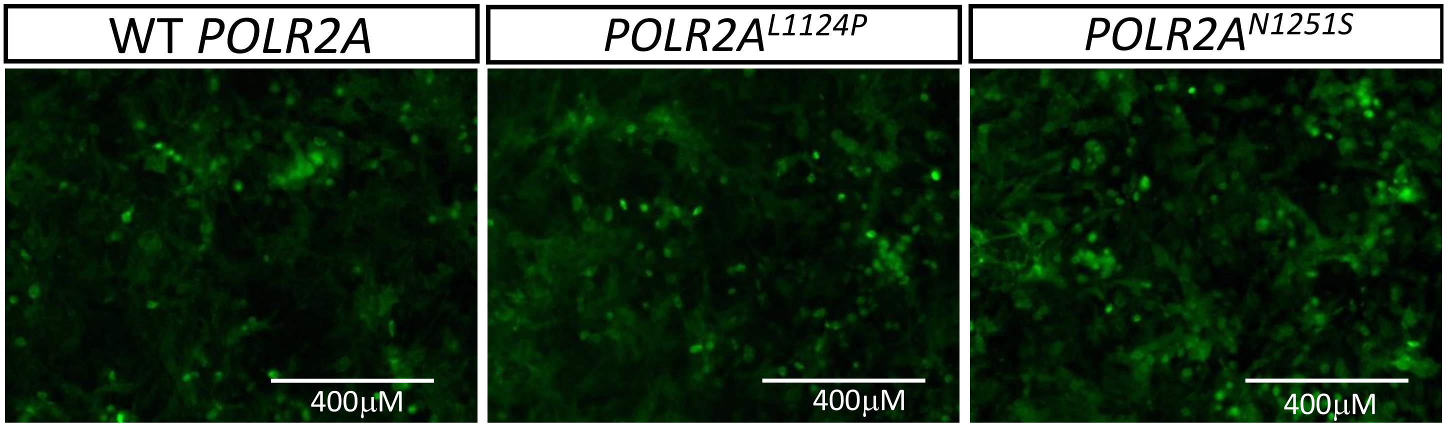

Figure S9 | Mutant and WT versions of *POLR2A* were tagged with EGFP and expressed ectopically from a plasmid. Although expression of all POLR2A versions was high, some POLR2A protein did not seem to enter the nucleus. Differences in the amount of protein that did not enter the nucleus between mutant versions could explain the relatively small increase in transcription errors in *POLR2A*^{L1124P} cells compared to *POLR2A*^{N1232S} cells. This experiment was performed twice.

Figure S10

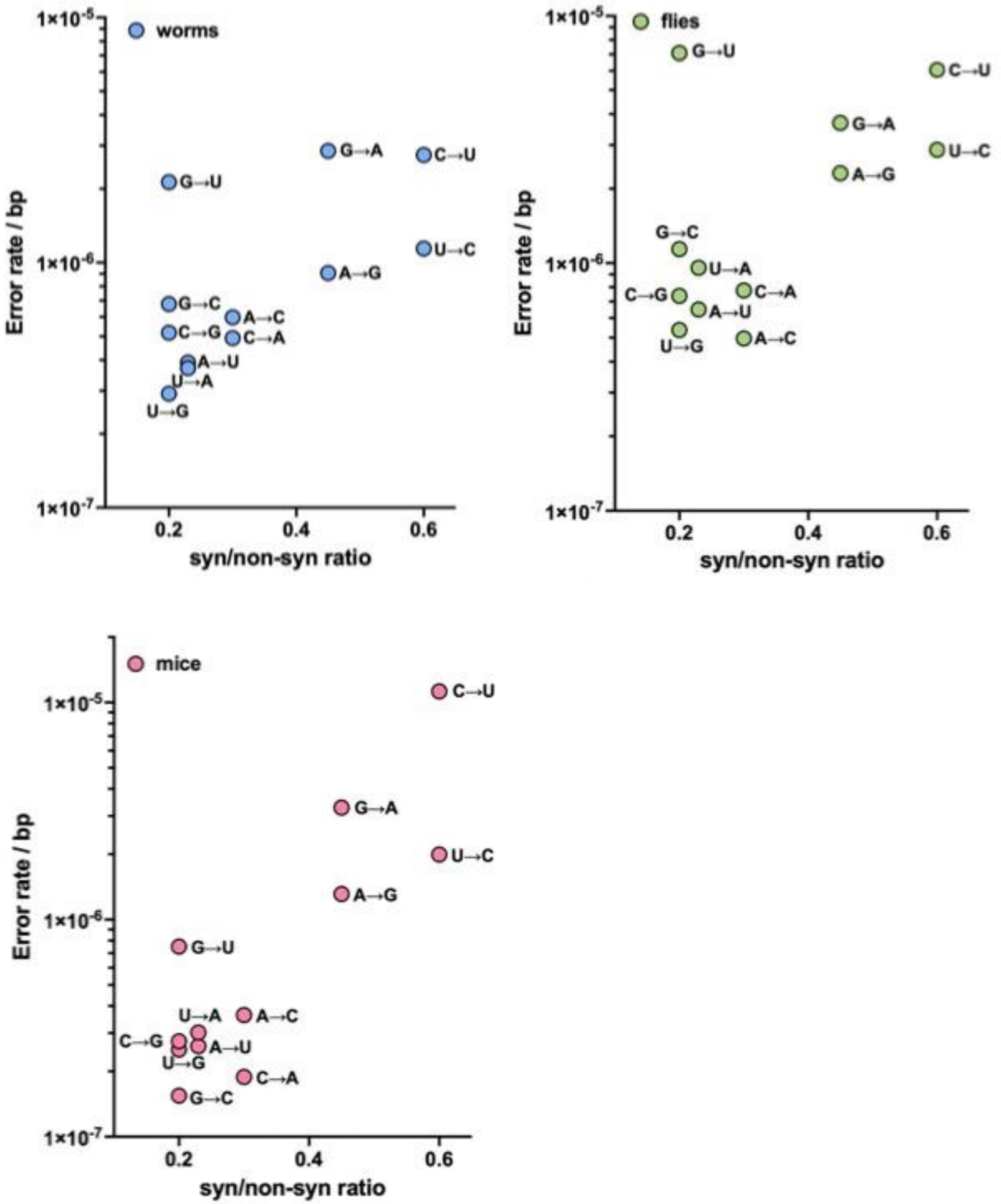

worms
flies
mice
Error rate / bp
syn/non-syn ratio
1×10-5
1×10-6
1×10-7
0.2
0.4
0.6
G→A
C→U
G→U
U→C
A→G
G→C
A→C
C→G
C→A
A→U
U→A
U→G

Figure S10 | The error rate of transcription and the impact of errors on protein coding sequences. To test whether there is a relationship between transcription errors and the genetic code, we expressed the error rate of transcription as a function of the chance that an error results in a synonymous or non-synonymous change. We found that higher the chance that an error results in a synonymous change (as expressed by the synonymous/non-synonymous ratio), the higher the error rate of that error type is. Conversely, the higher the chance that an error results in a non-synonymous change, the lower the error rate is.
