# Supplementary material for: Evolutionary conservation of the fidelity of transcription
Source: Nat Commun. 2023 Mar 20;14:1547. doi: 10.1038/s41467-023-36525-w (PMC10027832; doi:10.1038/s41467-023-36525-w)
Supplement: Supplementary file 2 — Reporting Summary [file 41467_2023_36525_MOESM2_ESM.pdf]

nature portfolio

Corresponding author(s): Marc Vermulst
Last updated by author(s): Jan 10, 2023

Reporting Summary

Nature Portfolio wishes to improve the reproducibility of the work that we publish. This form provides structure for consistency and transparency in reporting. For further information on Nature Portfolio policies, see our Editorial Policies and the Editorial Policy Checklist.

Statistics

For all statistical analyses, confirm that the following items are present in the figure legend, table legend, main text, or Methods section.

n/a	Confirmed	
☐	☒	The exact sample size (*n*) for each experimental group/condition, given as a discrete number and unit of measurement
☐	☒	A statement on whether measurements were taken from distinct samples or whether the same sample was measured repeatedly
☐	☒	The statistical test(s) used AND whether they are one- or two-sided<br>*Only common tests should be described solely by name; describe more complex techniques in the Methods section.*
☒	☐	A description of all covariates tested
☒	☐	A description of any assumptions or corrections, such as tests of normality and adjustment for multiple comparisons
☐	☒	A full description of the statistical parameters including central tendency (e.g. means) or other basic estimates (e.g. regression coefficient) AND variation (e.g. standard deviation) or associated estimates of uncertainty (e.g. confidence intervals)
☐	☒	For null hypothesis testing, the test statistic (e.g. *F*, *t*, *r*) with confidence intervals, effect sizes, degrees of freedom and *P* value noted<br>*Give P values as exact values whenever suitable.*
☒	☐	For Bayesian analysis, information on the choice of priors and Markov chain Monte Carlo settings
☒	☐	For hierarchical and complex designs, identification of the appropriate level for tests and full reporting of outcomes
☒	☐	Estimates of effect sizes (e.g. Cohen's *d*, Pearson's *r*), indicating how they were calculated

Our web collection on statistics for biologists contains articles on many of the points above.

Software and code

Policy information about availability of computer code

Data collection	no software was used for data collection.
Data analysis	Blast+ v. 2.13.0, homologene (https://www.ncbi.nlm.nih.gov/homologene/), Prism (v. 9.4.0) and custom software to analyze circ-seq datasets can be found at GitHub: https://github.com/jfgout/circseq-seqan2

For manuscripts utilizing custom algorithms or software that are central to the research but not yet described in published literature, software must be made available to editors and reviewers. We strongly encourage code deposition in a community repository (e.g. GitHub). See the Nature Portfolio guidelines for submitting code & software for further information.

Data

Policy information about availability of data

All manuscripts must include a data availability statement. This statement should provide the following information, where applicable:

- Accession codes, unique identifiers, or web links for publicly available datasets
- A description of any restrictions on data availability
- For clinical datasets or third party data, please ensure that the statement adheres to our policy

The sequencing data obtained for this study can be accessed at the SRA website (https://www.ncbi.nlm.nih.gov/sra) under accession codes: PRJNA924347, PRJNA923732, PRJNA923308, PRJNA922590, PRJNA672117, PRJNA673738, PRJNA673853, and PRJNA673744
There are no restrictions to data availability

Human research participants

Policy information about studies involving human research participants and Sex and Gender in Research.

Reporting on sex and gender	N/A
Population characteristics	N/A
Recruitment	N/A
Ethics oversight	N/A

Note that full information on the approval of the study protocol must also be provided in the manuscript.

Field-specific reporting

Please select the one below that is the best fit for your research. If you are not sure, read the appropriate sections before making your selection.

- [x] Life sciences
- [ ] Behavioural & social sciences
- [ ] Ecological, evolutionary & environmental sciences

For a reference copy of the document with all sections, see nature.com/documents/nr-reporting-summary-flat.pdf

Life sciences study design

All studies must disclose on these points even when the disclosure is negative.

Sample size	Sample size for circle-sequencing experiments has been empirically determined from previous studies in which the impact of different genes and conditions were assessed (Fritsch et al. Science Advances 2017)
Data exclusions	No data was excluded from this study
Replication	Multiple experiments contained additional biological replicates of untreated or WT samples, which served to increase the size of our total replicate pool, and as a standard to ensure accuracy of our assays. In each case, these additional samples yielded highly similar results that were indistinguishable from previous replicates performed weeks, months or years earlier. In addition, multiple samples were re-sequenced in order to acquire more data, improve statistical power or fleshing out error spectra. These resequencing efforts were always indistinguishable from initial measurements.
Randomization	The experiments were not purposefully randomized. However, all experiments were performed at the bench by multiple investigators. The circle-sequencing protocol takes 16-24 hours to complete over a 2-day timespan and requires multiple researchers to process various stages. During these stages, samples are labeled by numbers and divided over 2 or three researchers, each of which receives one or two of the replicates for a certain condition or genotype, so that all replicates are the result of at least 2 pairs of hands. No efforts were made to control the order in which the samples were processed.
Blinding	Although the investigators were not technically blinded to allocation during experiments and outcome assessment, all bench work experiments were performed at USC and UPenn, and analyses of sequencing data from these experiments were performed by a second, remote team located in Mississippi State who were usually not familiar with the details of the samples that were sequenced and were not part of the detailed design of the experiments themselves

Reporting for specific materials, systems and methods

We require information from authors about some types of materials, experimental systems and methods used in many studies. Here, indicate whether each material, system or method listed is relevant to your study. If you are not sure if a list item applies to your research, read the appropriate section before selecting a response.

Materials & experimental systems

n/a	Involved in the study	
x		Antibodies
	x	Eukaryotic cell lines
x		Palaeontology and archaeology
	x	Animals and other organisms
x		Clinical data
x		Dual use research of concern

Methods

n/a	Involved in the study	
x		ChIP-seq
x		Flow cytometry
x		MRI-based neuroimaging

Eukaryotic cell lines

Policy information about cell lines and Sex and Gender in Research

Cell line source(s)	HEK293 cell lines were purchased from ATCC: CRL-1573
Authentication	Cell lines were sequenced and observed by microscopy to authenticate them
Mycoplasma contamination	Cell lines were negative for mycoplasma contamination
Commonly misidentified lines (See ICLAC register)	No commonly misidentified cell lines were used in this study

Animals and other research organisms

Policy information about studies involving animals; ARRIVE guidelines recommended for reporting animal research, and Sex and Gender in Research

Laboratory animals	Canton-S Drosophila melanogaster (10 day old and 60 day old) and recently hatched N2 Caenorhabditis elegans were used in this study
Wild animals	no wild animals were used in this study
Reporting on sex	Only male Canton-S flies were used in this study, the Caenorhabditis elegans is hermaphroditic
Field-collected samples	No field collected samples were used in this study
Ethics oversight	This study did not require ethical approval

Note that full information on the approval of the study protocol must also be provided in the manuscript.
